# Woodchuck and deer Hepatitis Delta-like agents show distinct innate immune activation and IFN-resistance compared to human Hepatitis D Virus

**DOI:** 10.1038/s41598-026-45998-w

**Published:** 2026-04-01

**Authors:** Gnimah Eva Gnouamozi, Annick Charlotte Kooij, Marie Rose Schrimpf, Benno Zehnder, Zhenfeng Zhang, Stephan Urban

**Affiliations:** 1 Heidelberg University, Medical Faculty Heidelberg, Department of Infectious Diseases, Molecular Virology, Heidelberg, Germany; 2 School of Public Health and Emergency Management, School of Medicine, Southern University of Science and Technology, Shenzhen, China; 3 German Center for Infection Research (DZIF), Partner Site Heidelberg, Heidelberg, Germany

**Keywords:** HDV, HDV-like agents, Cell division-mediated spread, Innate immunity, Interferon, Viral evolution, Viral persistence, Virology, Viral evolution, Viral immune evasion

## Abstract

Replication of human hepatitis delta virus (HDV) is sensed by the pattern recognition receptors (PRRs) melanoma differentiation antigen 5 (MDA5) and Laboratory of Genetics and Physiology 2 (LGP2), activating interferon (IFN) responses. Both HDV-induced IFNs and exogenously administered IFN-α and -λ suppress HDV RNA spread via cell division (CDMS) but have only minor effects on replication in resting cells. With the discovery of HDV-like agents (DLAs) in diverse animal species an opportunity arose to comparatively study the interplay between these evolutionarily related replicons with respect to innate immune responses. To overcome the lack of authentic *in vitro* infection models for the viroid-like agents, we established a Hepatitis B virus (HBV)-envelope protein (HBsAg) pseudotype-based infection system for woodchuck (WoDV) and deer (DeDV) DLA. Infection of human hepatic cell lines with WoDV/HBsAg and DeDV/HBsAg pseudo-typed virions allowed initiation of replication in HuH7^NTCP^ cells. To assess the interdependence of the innate immune system and viral persistence, innate immunocompetent HepaRG^NTCP^ cells were infected with HDV, WoDV/HBsAg and DeDV/HBsAg. Consistent with previous findings, HDV replication robustly activated IFN responses, while replication of WoDV and, to a lesser extent, DeDV induced weaker IFN responses, as measured by IFN stimulated gene expression. Moreover, while CDMS of HDV was abrogated by IFN (either endogenous activation or exogenous administration), CDMS of WoDV and DeDV was not majorly affected, indicating different resistance mechanisms to IFN treatment, compared to HDV. Importantly, this phenotype of IFN resistance during cell division was confirmed for WoDV in woodchuck-derived hepatoma cells (WCH17^NTCP^), a model closer to the natural host. Furthermore, we demonstrated that the large delta antigen of HDV does not contribute to interferon sensitivity during cell division, suggesting that other viral or cellular factors mediate this process. Together, these results revealed that non-human DLAs display immune evasion strategies distinct from HDV, providing new insights into deltavirus biology and its evolutionary diversity.

**Supplementary Information:**

The online version contains supplementary material available at 10.1038/s41598-026-45998-w.

## Introduction

Hepatitis D virus (HDV) is a unique satellite virus that requires hepatitis B virus (HBV) for virion formation, and co-infection leads to the most severe form of viral hepatitis. Its clinical significance lies in its association with rapid progression to liver cirrhosis, hepatocellular carcinoma, and increased mortality compared to HBV infection alone.

HDV can spread extracellularly through packaging its ribonucleoprotein complex (RNP) into self-assembly competent HBV envelope proteins (HBsAg) allowing specific *de novo* infection of sodium taurocholate co-transporting polypeptide (NTCP)-expressing hepatocytes. Alternatively, HDV can spread its RNA through cell division, which has been shown in cell culture and the human liver chimeric mouse model. This latter spreading pathway is independent of HBsAg expression^1,2^. The phenotype of this intracellular spread *in vitro* is the formation of clusters of hepatitis delta antigen (HDAg)-positive cells in the absence of HBsAg indicating the transmission of HDV RNA from mother to daughter cells, via clonal expansion.

While cell division-mediated spread (CDMS) has been demonstrated *in vitro* and in immune-incompetent mouse models, direct confirmation in patients is lacking. Indirect evidence may come from liver transplant studies, where HDAg staining persisted despite the absence of HBV markers, and from the higher efficacy of combination therapy with interferon (IFN) and bulevirtide (BLV), which may suggest a role of CDMS in persistence^3,4^.

In contrast to HBV, which is regarded as a stealth virus that does not induce significant IFN responses during *in vitro or in vivo* infection^5–7^, HDV triggers the induction of IFNs (ß and λ) and consequently mounts a cellular antiviral state. HDV replication is sensed via the recognition of viral RNA by the two pattern recognition receptors (PRRs) melanoma differentiation antigen 5 (MDA5)^8^ and Laboratory of Genetics and Physiology 2 (LGP2)^9^.

Human hepatoma-derived HuH7 cells are widely used for HBV and HDV studies due to their ease of culture and permissiveness upon NTCP expression, but they are deficient in key innate immune signaling pathways, including the RIG-I–like receptor axis, and therefore mount only a limited IFN response^10^.In contrast, HepaRG cells retain functional PRR expression and downstream signaling, enabling robust induction of IFN-stimulated genes (ISGs) upon double-stranded (ds) RNA sensing^11^. This difference is important for the present study, as HepaRG cells provide an immunocompetent model to assess innate immune activation by HDV and HDV-like agents (DLA), whereas HuH7 cells serve as a permissive immune-deficient comparison.

However, how recognition of HDV replication occurs remains to be characterized. HDV replicates in the nucleus of infected cells, but the PRRs that sense the HDV RNA intermediates predominantly localize in the cytoplasm^12^.

One hypothesis is that a small fraction of MDA5 may localize in the nucleus, allowing sensing of HDV replication. Or the HDV RNP complexes are transported to the cytoplasm where they are captured by PRRs^13^. Previous studies have demonstrated that HDV RNA is present in both the nucleus and the cytoplasm, and that HDV RNP complexes undergo active nucleocytoplasmic shuttling. *Tavanez et al.* showed by heterokaryon assays that HDV RNPs continuously move between the two compartments, with the small hepatitis delta antigen (S-HDAg) mediating nuclear import of viral RNA and facilitating its subsequent export^14^. In the absence of delta antigen, HDV RNA accumulates in the cytoplasm, whereas co-expression of S-HDAg drives nuclear localization^15^. Kinetic studies further revealed transient export of newly synthesized HDV RNA to the cytoplasm followed by reimport into the nucleus^16^.These findings support the model that HDV RNA intermediates can be exposed to cytoplasmic PRRs, providing a mechanistic basis for innate immune sensing.

Although the induction of innate immune responses by HDV replication triggers the production of IFNs, these IFNs have only a limited effect on HDV replication in resting cells of hepatic origin^8,17^. In contrast, HDV-induced IFN responses as well as exogenous IFN treatment strongly restrict the efficacy of CDMS of HDV RNA. So far, the mechanism (e.g. the specific ISGs involved in RNA degradation) of this counteraction remains elusive. One hypothesis is that during mitosis, nuclear membrane disruption could expose the HDV replication intermediates to PRRs, thereby rendering the viral RNA susceptible to effectors that subsequently promote degradation^17^.

In the last years, DLA were first identified through metagenomic studies that revealed similar circular RNA genomes in a variety of non-human species, including birds, snakes, fish, and invertebrates^18–23^. The identification of HDV-like agents in non-human species led to the creation of a new virus family, the Kolmioviridae. The designation “Kolmio,” taken from the Finnish word for triangle, alludes to the Greek letter Δ (delta), which represents the genus Deltavirus within the realm Ribozyviria^24^. These agents share structural and functional characteristics with human HDV, such as the presence of ribozymes and a delta antigen-like protein. Besides the snake delta agent (SDeV), discovered in boa constrictors co-infected with reptarenaviruses and able to be packaged by their envelope proteins to form infectious particles *in vitro*^25,26^, no specific helper viruses of DLAs have been identified so far. Indeed, in contrast to HDV, which relies on HBV-encoded envelope proteins for propagation via virions, DLAs do not depend on hepadnaviral envelope proteins. This is reflected by the absence of a genetically encoded intrinsic large delta antigen (L-DAg) expression^20,27^, which is essential in HDV life cycle as key determinant for HDV RNP packaging by HBsAg. Among these, a woodchuck delta-like agent (WoDV) and a white-tailed deer delta-like agent (DeDV) were discovered in transcriptomic datasets^22,28^. Both agents share key genomic and structural features with HDV, such as a small, circular, negative-sense RNA genome with a self-complementary rod-like structure and the ability to encode a delta antigen-like protein. However, infectious virions have not yet been isolated from natural hosts, and packaging by HBsAg was reported inefficient by 2 independent studies^20,27^.

However, both HDV and DLAs can propagate via CDMS^23,27^. So far, only a few studies are addressing the interaction between DLAs and the host innate immune system. It would be tempting to speculate that CDMS may play an important role in their replication, persistence, and that possible counteraction by cellular innate immune responses must be evaded for this process. *Paraskevopoulou et al.*. evaluated the immune response in *P. semispinosus*, the original host of the rodent DLA (RDeV)^23^. Both RDeV RNA-positive and RDeV RNA-negative serum samples tested positive for anti-RDeAg antibodies, similar to HDV infection in humans. Another study took advantage of hydrodynamic delivery of cDNA of the RDeV genome into an *in vivo* mouse model, showing ISG upregulation^29^. Studying the induction of innate immunity upon infection with DLAs may play a critical role in better understanding host-to-host transmission and putative evasion mechanisms from the immune system. In the present work we investigate IFN induction and regulation of woodchuck and deer DLA in human and non-human derived cell lines. Our data highlight potential evolutionary differences between HDV and DLAs regarding their activation and sensitivity to the IFN response. Moreover, the difference observed in animal versus human delta virus raises the question whether possible differences between the eight human HDV genotypes may play a role in clearance rates and sensitivity to IFN therapy.

## Results

### HuH7^NTCP^ and HepaRG^NTCP^ cells support WoDV and DeDV replication following infection with HBsAg-pseudotyped particles

**Fig. 1:**
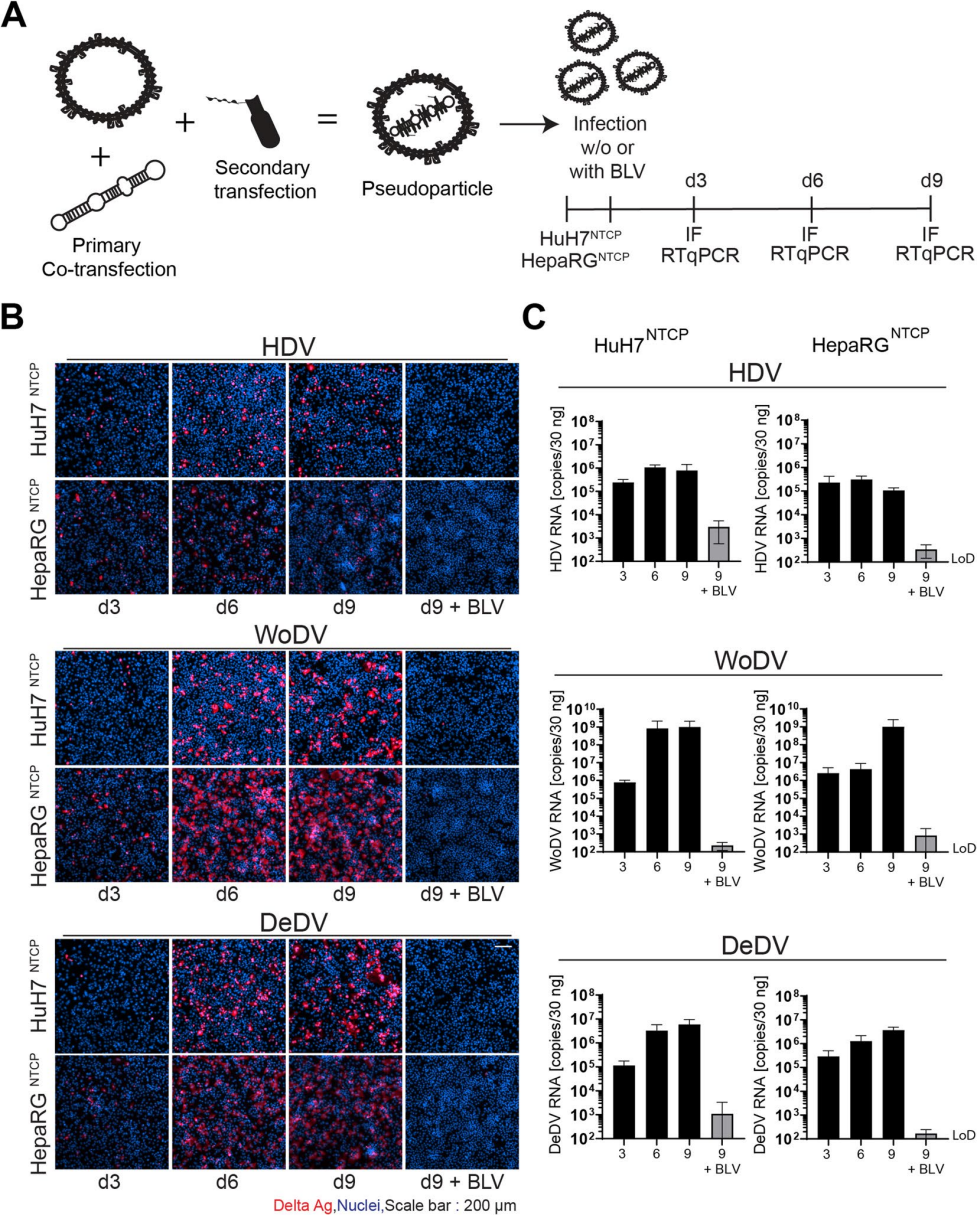
Replication kinetics of HDV, WoDV, and DeDV in human hepatic-derived cell lines. **(A)** Pseudoparticles were generated in HuH7 cells by co-transfection with plasmids encoding HDV or DLA (WoDV, DeDV) RNA (1.1x genome) and HBsAg (genotype D), followed three days later by trans-complementation with an L-HDAg plasmid to allow RNP packaging. Viral supernatants were harvested, purified by heparin-Sepharose chromatography, and used to infect HuH7^NTCP^ and HepaRG^NTCP^ cells (MOI = 1). **(B)** Infected cells were fixed at days 3, 6, and 9 post-infection (pi) and stained by immunofluorescence (IF) using monoclonal antibody FD3A7 (Kerafast, Cat# EHD001, 1:3000) recognizing HDAg/WoDAg/DeDAg, followed by Alexa Fluor 546-conjugated secondary antibody. Nuclei were counterstained with Hoechst 33342. Representative images are shown from three independent experiments. Scale bar: 200 μm. **(C)** Intracellular viral RNA was quantified by RT-qPCR at days 3, 6, and 9 pi, normalized to GAPDH, and displayed compared to BLV-treated control at days 9 pi (200 nM BLV). Values represent mean ± SD of biological duplicates with technical triplicates.

Human hepatocellular carcinoma cells (HuH7) were co-transfected with plasmids encoding a 1.1-fold over-length antigenome of HDV (construct pJC126, genotype 1, provided by Dr. John Taylor), WoDV, or DeDV DLA^27^, and a pT7HB2.7 vector encoding for HBsAg (HBV genotype D^30^. In addition, a plasmid encoding the large hepatitis delta antigen (L-HDAg) was trans-complemented to allow packaging of the RNP complex by HBsAg. This step is crucial for pseudotyping and viral particle formation of WoDV and DeDV since they lack the expression of a genuine L-DAg (Fig.S1) and, therefore cannot be naturally enveloped by HBsAg^20,27^. Infectivity of viral pseudoparticles in collected supernatants was assessed by titration of dilution series in NTCP-expressing HuH7 cells (Fig.S2). To characterize replication kinetics upon a synchronized receptor-mediated binding and internalization, two human hepatic cell lines, HuH7^NTCP^ and HepaRG^NTCP^ were inoculated with HDV, WoDV, or DeDV pseudoparticles, and either fixed for viral antigen (DAg) immunostaining or harvested for intracellular viral RNA quantification via RT-qPCR on day 3, 6 or 9 post infection (pi) (Fig. 1A). In both HuH7^NTCP^ and HepaRG^NTCP^ cells, HDV and the HBsAg-pseudotyped DLAs established replication, as indicated by an increase of DAg-positive cells (Fig. 1B) and an increase of intracellular viral RNA from day 3 to day 6 pi (Fig. 1C). Human HDV showed a plateau for both DAg expression and RNA already at day 6 pi, while WoDV and DeDV RNA further increased until 9 days pi. Discrepancy between antigen expression and RNA level for WoDV could be due to differences in RNA stability or antigen accumulation between cell types.

Infection was abrogated by the entry inhibitor BLV indicating an NTCP-dependent infection of all three DLAs.

### HDV, WoDV and DeDV induce innate immune responses at different levels in HepaRG^NTCP^ cells

**Fig. 2:**
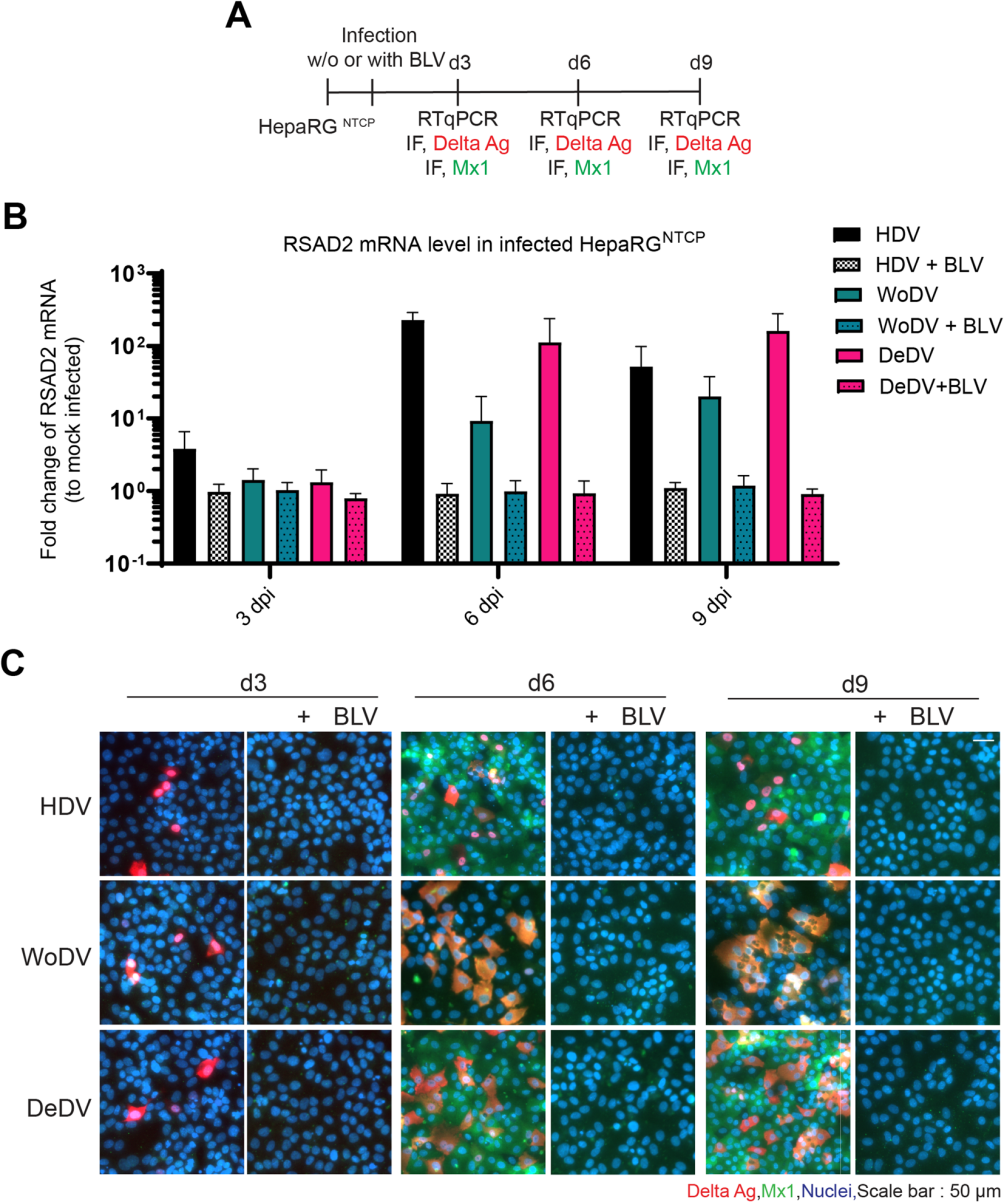
Innate immune responses induced by HDV, WoDV, and DeDV in HepaRG^NTCP^ cells. **(A)** HepaRG^NTCP^ cells were infected with pseudoparticles carrying HDV, WoDV, or DeDV genomes (MOI = 1). BLV (200 nM) was included in parallel conditions as entry control. **(B)** At days 3, 6, and 9 pi, RSAD2 mRNA levels were quantified by RT-qPCR, normalized to GAPDH, and expressed relative to mock-infected cells. Data represent mean ± SD of biological duplicates with technical triplicates. **(C)** IF staining for viral antigens (FD3A7 antibody, as in Fig. 1) and Mx1 (Santa Cruz, Cat# sc-271527, 1:200) was performed at days 3, 6, and 9 pi. Nuclei were counterstained with Hoechst 33342. Representative images are shown from three independent experiments. Scale bar: 50 μm.

Given the establishment of viral replication upon pseudotyped DLAs infection, we investigated infections of the three agents at comparable multiplicity of infection (MOI). Innate immune competent HepaRG^NTCP^ cells were infected with equivalent MOI of HDV, WoDV or DeDV pseudoparticles and harvested at day 3, 6 or 9 pi (Fig. 2A), as part of the same experiment shown in Fig. 1. Following cellular RNA extraction, we quantified IFNs and ISGs expression via RT-qPCR. RSAD2 was chosen as representative for ISG induction, since it has been shown previously to be upregulated during HDV infection^8,31^. As control, cells were inoculated with pseudoparticles in the presence of BLV to evaluate possible effects caused by the inoculum. Although WoDV and DeDV established a robust infection in HepaRG^NTCP^, as shown by DAg staining of infected cells, the induction of RSAD2 at day 6 pi was lower for both DLAs compared to HDV (Fig. 2B). Moreover, RSAD2 expression increased until day 6 pi, with no major increase at day 9. To validate the findings from bulk ISG measurements, the upregulation of ISGs was also assessed through staining of Mx1 in individual cells in the absence and presence of BLV. While viral DAgs were only detected from day 3 onwards in infected HepaRG^NTCP^ cells in the absence of BLV, Mx1 staining was only observed starting on day 6 pi, suggesting that Mx1 induction required viral RNA replication (Fig. 2C). Mx1 expression was detectable in HDV-infected HepaRG^NTCP^ cells reaching a peak at day 6 pi. Mx1 upregulation was observed in DeDV-infected cells at day 6 and 9 pi. In contrast, WoDV infection resulted in a weak Mx1 expression which correlated with the weak induction of RSAD2 determined by RT-qPCR. WoDV and DeDV infection rates still increased at day 9 pi, while HDV reached a plateau at day 6 pi. Notably, Mx1 induction was not only observed in infected cells, but also in DAg-negative cells that surrounded infected cells, as its overexpression can be induced in bystander cells through paracrine IFN signaling. Infection of HepaRG^NTCP^ MDA5 knockout cells revealed a central role for this PRR in sensing DeDV replication, as the loss of MDA5 completely abolished RSAD2 upregulation in DeDV-infected cells (Fig. S10).

Considering the difference in the intensity of IFN activation upon HDV and DLAs infection, we further evaluated the impact of this phenotype in the context of viral CDMS.

### WoDV and DeDV can spread via cell division in both HuH7^NTCP^ and HepaRG^NTCP^ cells

**Fig. 3:**
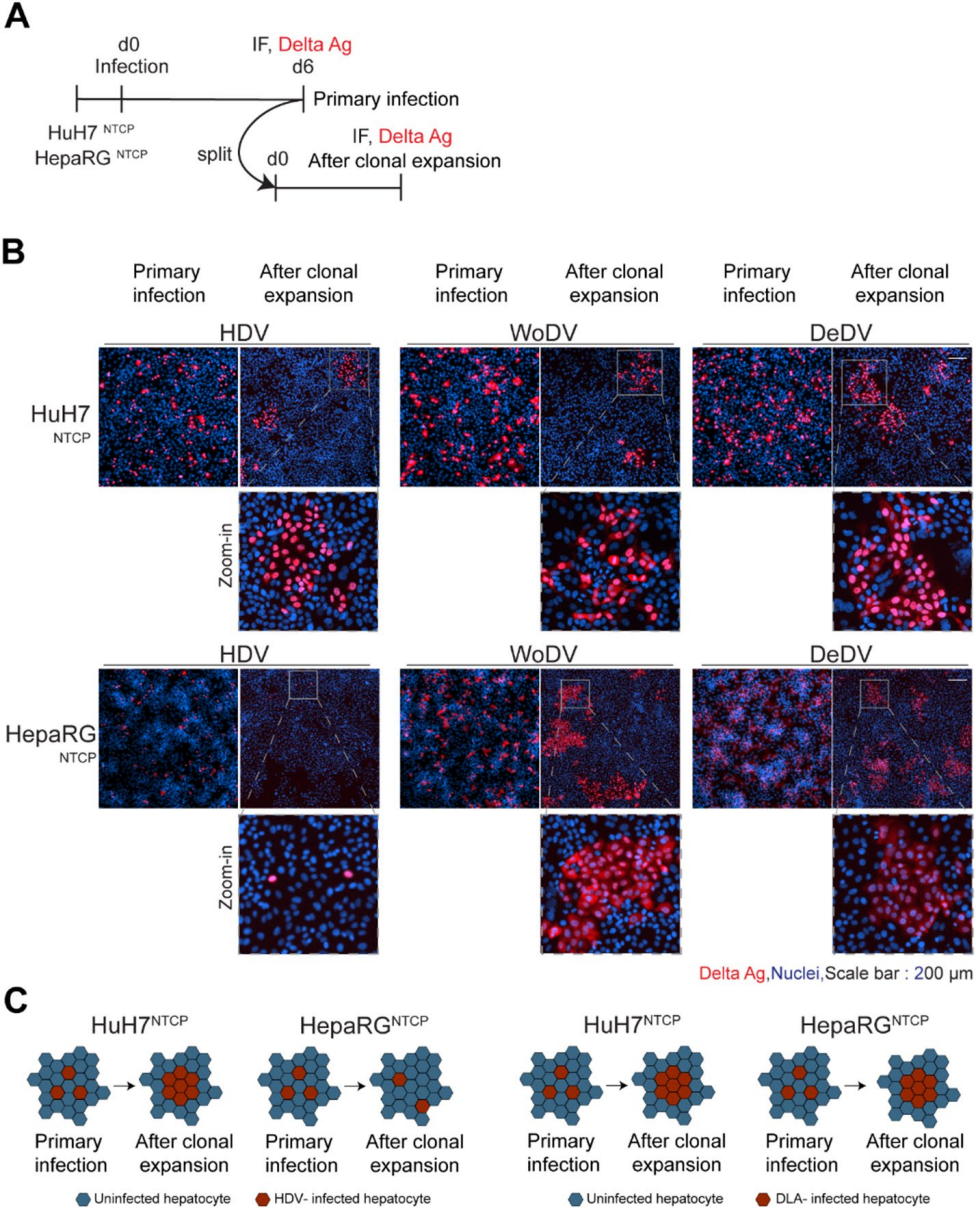
Cell division-mediated spread (CDMS) of HDV, WoDV, and DeDV in HuH7^NTCP^ and HepaRG^NTCP^cells. **(A)** Cells were infected with HDV, WoDV, or DeDV pseudoparticles (MOI = 1). At day 6 pi, cells were split at high dilution (1:100 for HuH7^NTCP^, 1:50 for HepaRG^NTCP^) to allow clonal expansion. **(B)** After reaching confluence, cells were fixed and stained with FD3A7 antibody for DAg visualization. Nuclei were counterstained with Hoechst 33342. Representative IF images are shown from three independent experiments. Scale bar: 200 μm. **(C)** Schematic representation of CDMS patterns observed for HDV and DLA in HuH7^NTCP^ and HepaRG^NTCP^ cells.

HDV can spread intracellularly allowing the persistence of viral RNA in the absence of HBV envelope proteins during mitosis, resulting in the formation of clusters of HDV-positive cells originating from a single infected “mother cell”^1,2^. In our previous work, we have shown that this spreading pathway also holds true for WoDV and DeDV^27^. To examine the impact of differential innate immunity induction on CDMS, innate immune incompetent (HuH7^NTCP^) and competent (HepaRG^NTCP^) cells were infected with either HDV, WoDV, or DeDV pseudoparticles. At day 6 pi cells were split and reseeded at a high dilution factor (1:100 and 1:50 for HuH7^NTCP^ and HepaRG^NTCP^, respectively) and further cultured to allow clonal expansion (Fig. 3A). As expected from previous studies, HDV but also WoDV and DeDV spread without restriction via cell division in innate immune incompetent HuH7^NTCP^ cells as indicated by the formation of large clusters of DAg-positive cells (Fig. 3B upper panel). When looking at CDMS in innate immune competent HepaRG^NTCP^ cells infected with HDV, restricted CDMS was observed (Fig. 3B lower panel). In contrast, WoDV and DeDV CDMS remained largely unaffected (Fig. 3B&C). In the case of WoDV, this might be associated with the weaker ISG induction by this DLA as shown in Fig. 2B&C. Given these results, we investigated the impact of exogenous IFN treatment on CDMS.

### Interferon treatment does not inhibit cell division-mediated spread of WoDV and DeDV in HuH7^NTCP^ cells

**Fig. 4:**
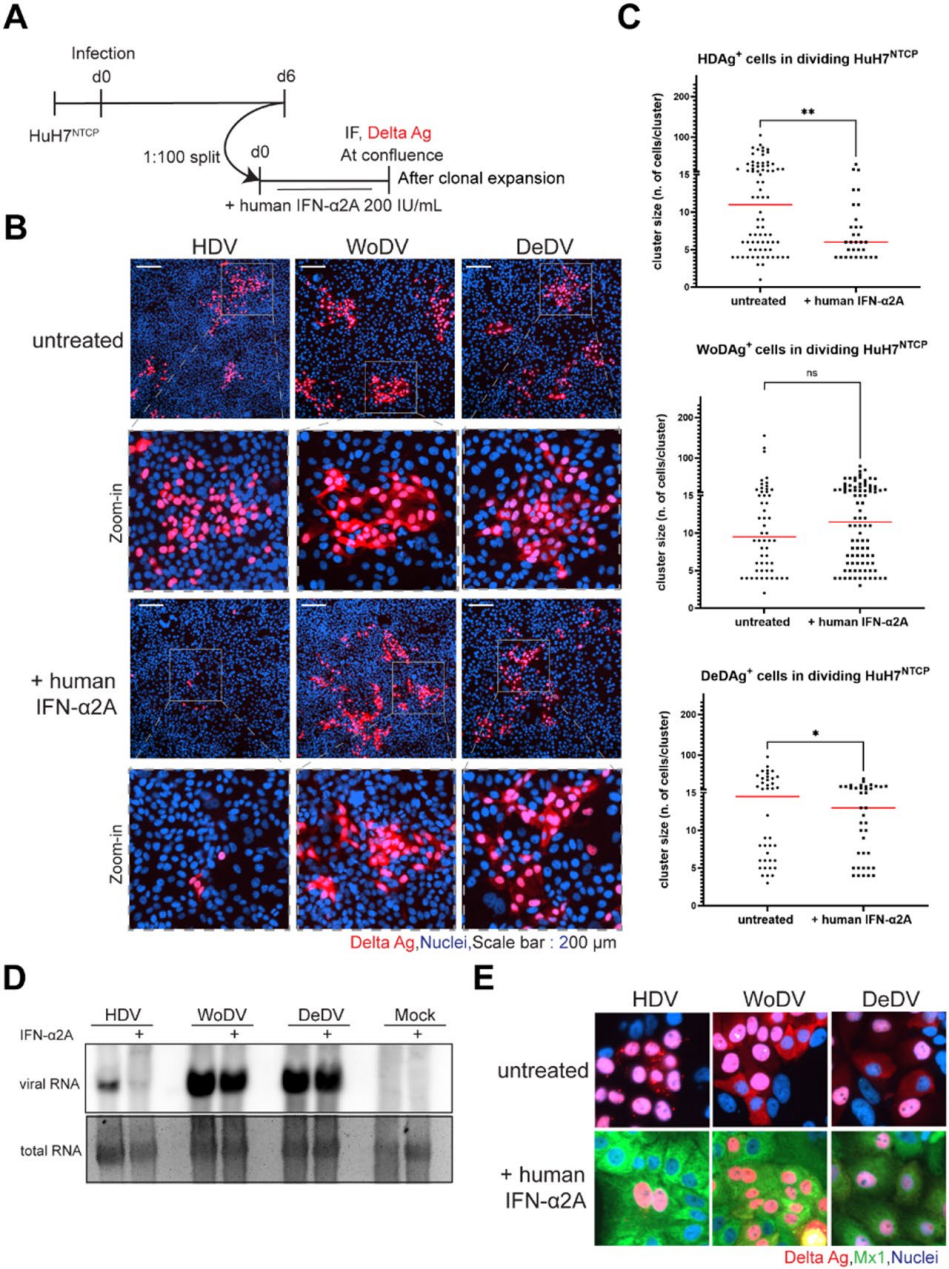
Effect of interferon treatment on cell division-mediated spread of WoDV and DeDV in HuH7^NTCP^ cells. **(A)** Experimental design: HuH7^NTCP^ cells were infected with HDV, WoDV, or DeDV pseudoparticles and passaged at day 6 pi (1:100 dilution). During clonal expansion, cells were treated with IFN-α2A (200 IU/mL). **(B)** Cells were fixed at confluence and stained with FD3A7 antibody for DAg. Representative fields are shown at low and higher magnification. Scale bar: 200 μm. **(C)** Clusters of DAg-positive cells were quantified using CellProfiler software; data represent mean ± SD from three independent experiments. **(D)** Viral RNA levels were assessed by Northern blotting with DIG-labeled probes specific for HDV, WoDV, or DeDV. Representative blot shown (uncropped version in Fig. S8). **(E)** IF co-staining for DAg (FD3A7 antibody) and Mx1 (Santa Cruz, Cat# sc-271527) was performed. Nuclei were counterstained with Hoechst 33342. Representative images from three experiments are shown. ns, not significant; *, *p* < 0.05; **, *p* < 0.01; ***, *p* < 0.001 (Student’s t test).

To investigate whether the unrestricted spread of WoDV and DeDV in HepaRG^NTCP^ cells was due to the observed weaker induction of IFNs by these two DLAs, we analyzed the sensitivity of CDMS against exogenously administered IFNs. To that end we used the HuH7^NTCP^ cell line that cannot mount an IFN response but is sensitive to exogenous IFNs, desplaying restricted HDV CDMS when treated with IFN-α and IFN-λ1^2^. HuH7^NTCP^ cells were infected, split on day 6 pi (dilution factor of 1:100), and IFN (IFN-α2A) was added to the culture medium (Fig. 4A). At confluence (day 6 post-splitting), formation of clusters of DAg-positive cells was analyzed. While human HDV lost the capability to spread upon IFN treatment (shown by HDAg staining), WoDV and DeDV presented extended clusters of DAg-positive cells (Fig. 4B). Clusters were quantified by counting the number of clusters and number of DAg+ cells for each cluster verifying a considerable reduction of DAg+ cells in HDV-infected HuH7^NTCP^ following treatment with IFN-α2A, but not for WoDV-infected or DeDV-infected cells (Fig. 4C). The impact of IFN treatment was also evaluated at the RNA level by northern blot analysis. In alignment with the IF results, a reduction in viral RNA was observed following clonal expansion solely for HDV. RNA levels of WoDV and DeDV exhibited only a slight decrease (Fig. 4D) indicating that RNA replication could proceed although the IFN pathway was activated. To determine if WoDV and DeDV DLAs played an active role in repressing innate immunity upregulation in infected cells, we performed co-staining of viral DAg and Mx1 (Fig. 4E). In contrast to HDV, CDMS of WoDV and DeDV was not affected even when Mx1 was upregulated in DAg-positive cells (Fig. 4E).

The same phenotype was observed for IFN-λ1 treatment in infected HuH7^NTCP^ cells during clonal expansion (Fig.S3). Taken together, our results indicate that both WoDV and DeDV are able to spread intracellularly upon IFN treatment in human hepatic cell lines.

### WoDV can persist via cell division in woodchuck-derived cells under IFN treatment

**Fig. 5:**
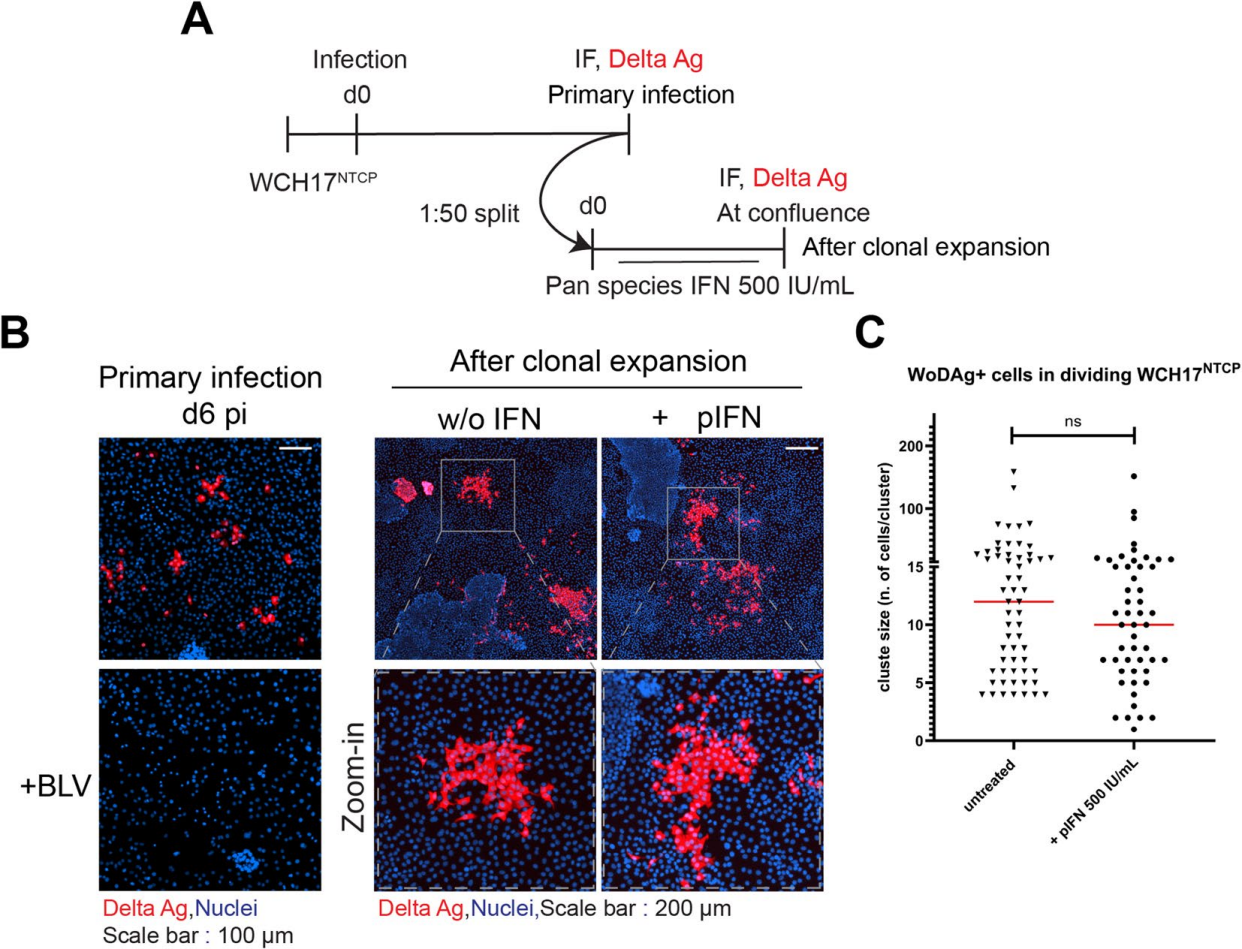
Cell division-mediated spread of WoDV in woodchuck hepatoma-derived WCH17^NTCP^ cells. **(A)** WCH17^NTCP^ cells were infected with WoDV pseudoparticles (MOI = 1) and stained for DAg at day 6 pi. **(B)** Left: infection rate was determined by IF (FD3A7 antibody). Right: in parallel, cells were split (1:100 dilution) and cultured in the presence of pan-species IFN-αA/D (500 IU/mL). After reaching confluence, clusters of DAg-positive cells were visualized. Nuclei were counterstained with Hoechst 33342. Scale bar: 200 μm. **(C)** Cluster number and size were quantified using CellProfiler. Data are displayed as mean ± SD from three independent experiments. Scale bar: 100 μm. ns, not significant; *, *p* < 0.05; **, *p* < 0.01; ***, *p* < 0.001 (Student’s t test).

Considering the phylogenetic distance between the host species in which the DLA were first identified and humans, we sought to investigate DLAs in a closer-to-original host model. To this aim we took advantage of the woodchuck (*Marmota monax*) hepatoma cell line WCH17^32^. Transfection of WCH17 cells with the dsRNA analog, Poly I: C, revealed that these cells possess only mild intrinsic immune recognition for dsRNA (Fig.S4A). However, the effect of exogenous IFN on the CDMS could be determined, as indicated by elicited IFN response upon treatment with a pan-species interferon (IFN-αA/D) (Fig.S4B). After transduction with NTCP (Fig.S4C), WCH17^NTCP^ cells were infected with WoDV pseudoparticles and infection rate was evaluated via IF staining at day 6 pi (Fig. 5A). HBV-pseudotyped WoDV infection of WCH17^NTCP^ cells proceeded in an NTCP-dependent manner, as shown by the sensitivity to BLV (Fig. 5B, left panel). In parallel, cells were split (1:50 dilution factor) and IFN-αA/D was added to stimulate innate immunity responses. At confluence, CDMS was visualized by DAg staining (Fig. 5B, right panel). Similar to human-derived hepatoma cell lines treated with human IFN, pan-species IFN treatment did not inhibit CDMS of WoDV in WCH17^NTCP^ cells (Fig. 5B). Through quantification of the number and size of cluster of WoDAg+ cells (Fig. 5C) we found no significant differences between untreated and pan-species IFN-treated cells. In summary, these results show that WoDV RNA amplification by cell division is resistant to IFN treatment in both human and woodchuck hepatoma cells, indicating that the difference in IFN sensitivity between HDV and WoDV is not restricted to human cell lines.

### The role of viral antigens in interferon sensitivity

The S-HDAg of HDV is well established to localize predominantly in the nuclei of both transfected and infected cells, consistent with its role in supporting viral RNA replication. However, previous studies have shown that S-HDAg localization is dynamic and influenced by both viral and host factors. Nuclear accumulation is mediated by a canonical nuclear localization signal (NLS) within S-HDAg, but cytoplasmic distribution can also be observed under certain conditions, particularly during late stages of replication or in the presence of nuclear export signals^14,33–36^. In contrast, transfection and HBV-pseudotype-mediated infection showed both cytoplasmic and nuclear staining of WoDAg and DeDAg within most of the cells (Fig.S5). Considering that viral RNA replicates in the nucleus while MDA5 senses viral RNA in the cytosol, different distribution pattern of WoDAg and DeDAg compared to HDAg may play a role in RNA replication or immune recognition for the DLAs, e.g. by circumventing innate immunity recognition and preventing sensitivity to an antiviral state^27^.

To test this hypothesis, we generated HuH7 cell lines stably expressing WoDAg or DeDAg (HuH7/WoDAg and HuH7/DeDAg) (Fig.S6A). Following IFN-α2A treatment we evaluated the degree of Mx1 induction in comparison to parental HuH7 cells (Fig.S6B). Induction levels upon IFN treatment were comparable across all cell lines (Fig.S6C) indicating that WoDAg or DeDAg do not impair the IFN signaling.

**Fig. 6:**
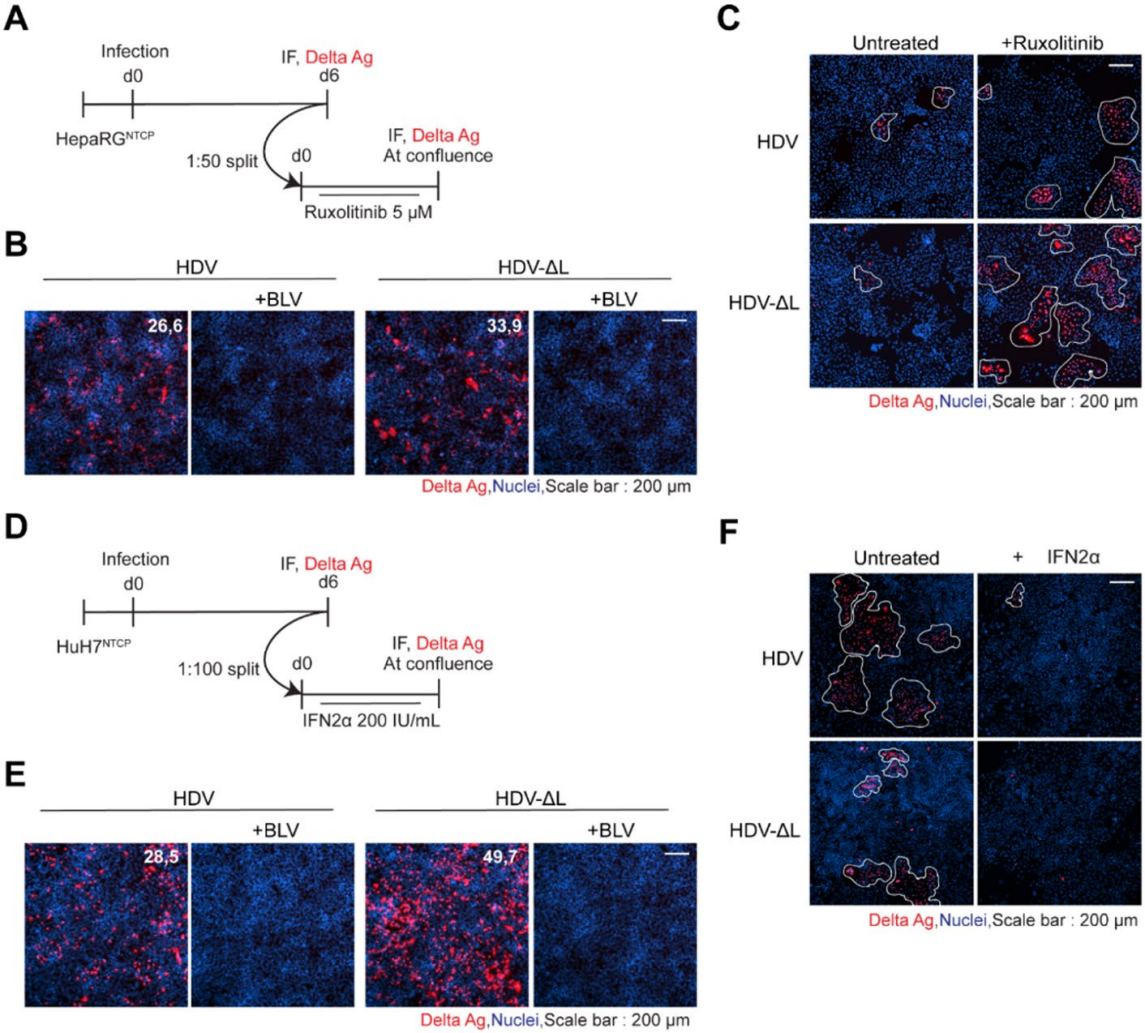
Effect of L-HDAg on interferon sensitivity of HDV. **(A**,** D)** HepaRG^NTCP^ **(A)** and HuH7^NTCP^ **(D)** cells were infected with wild-type (WT) HDV or an L-HDAg-deficient mutant (HDV-ΔL) . **(B**,** E)** At day 6 pi, infection rates were determined by IF staining for DAg (FD3A7 antibody). **(C**,** F)** Following high-dilution passage (1:50 for HepaRG^NTCP^, 1:100 for HuH7^NTCP^), cells were either untreated or treated with Ruxolitinib (5 µM) or IFN-α2A (200 IU/mL) and analyzed at confluence for CDMS by IF. Nuclei were counterstained with Hoechst 33342. Representative images from three independent experiments are shown. Scale bar: 200 μm.

Considering the inhibitory effect of L-HDAg in its farnesylated form on HDV replication and its connection to IFN signaling, we generated an HDV variant lacking L-HDAg expression (HDV-ΔL) (Fig.S7). We then evaluated the impact that the lack of L-HDAg expression has on replication and CDMS in infected innate immune-competent HepaRG^NTCP^ (Fig. 6A) and in innate immune-incompetent HuH7^NTCP^ (Fig. 6D). The infection rate at the chosen MGE at 6 days pi was higher for HDV-ΔL in both HepaRG^NTCP^ and HuH7^NTCP^ (Fig. 6B&E), due to the absence of L-HDAg-mediated self-restriction of replication^37,38^.

To confirm that CDMS inhibition of HDV and HDV-ΔL is dependent on innate immune signaling, we treated dividing HepaRG^NTCP^ cells with the JAK1/JAK2 inhibitor Ruxolitinib.

After high dilution passage in HepaRG^NTCP^ cells, a substantial reduction in CDMS for wild type (WT) HDV was observed. Likewise, CDMS of the HDV-ΔL variant remained affected in the innate immune competent HepaRG^NTCP^ cells, as indicated by the lack of cluster formation and only single HDAg positive cells (Fig. 6C). Analysis of CDMS showed that both agents propagated effectively in HepaRG^NTCP^ during treatment with the JAK1/JAK2 inhibitor, Ruxolitinib, and in HuH7^NTCP^ cells due to the lack of innate immunity induction. Consistent with these findings, WT HDV and the variant deficient in L-HDAg expression were not able to propagate replicating RNA via mitosis during IFN treatment (Fig. 6F). These findings suggest that L-HDAg was not a key factor determining HDV sensitivity to IFN during cell division.

## Discussion

This study aimed at investigating the interaction between two representative DLAs (DeDV and WoDV) and host cell innate immune responses in comparison to HDV. Through the establishment of an *in vitro* infection cell culture system, which relied on the construction of HBsAg-based pseudoparticles, we investigated the interplay between DLA and host innate immune responses. Artificial pseudotyping of the DLA RNPs enabled us to infect NTCP-expressing cells of woodchuck and human origin with a defined MOI, resulting in initiation of replication. Virions were generated by co-expression of HBV envelope proteins and trans-complementation of an L-HDAg expression plasmid. This methodology offered a significant advantage since it allowed investigating the impact of the absence of L-DAg or helper virus on viral replication, sensing, innate immune induction, and spread via cell division.

Infection of HuH7^NTCP^ and HepaRG^NTCP^ cells with WoDV and DeDV HBsAg-pseudoparticles resulted in the establishment of robust replication of the artificially packaged RNAs (Fig. 1) of both DLAs even in hepatic cell lines of human origin. This supported previous studies^20,27^ and indicated a very limited dependence on host specific factors, also shown previously for HDV, which is able to infect cells from several non-human species upon expression of human NTCP^39,40^. Remarkably, WoDV replicated at even higher levels, when compared to HDV. Although showing higher replication, WoDV and DeDV infections lead to a lower ISG induction when compared to HDV (Fig. 2). Moreover, both DLAs showed no major difference in restriction of RNA spread via cell division between innate immune incompetent HuH7^NTCP^ and innate immune competent HepaRG^NTCP^ (Fig. 3). Furthermore, and in sharp contrast to HDV, the CDMS of WoDV and DeDV RNAs were not affected by IFN treatment in infected HuH7^NTCP^ cells (Fig. 4).

As viruses adapt to host cells, they evolve mechanisms to escape or partially counteract viral recognition aiming at host-mediated clearance^41–43^. Since WoDV and DeDV replicate in hosts not closely related to humans, differences in the innate immune systems may be critical for proper recognition and for counteracting infection. Considering this aspect, we investigated CDMS of woodchuck DLA in a woodchuck-derived hepatoma cell line, during exogenous IFN treatment. We showed that, despite activation of the innate IFN response, CDMS of WoDV RNA was barely affected, suggesting that WoDV evolved a mechanism to actively counteract IFN-mediated repression of spread during cell proliferation, even in woodchuck hepatoma cells (Fig. 5). Whether this also holds true for DLAs of other origins in their respective host cells requires further investigation. Since it is still unclear whether WoDV naturally replicates in the liver, identifying the primary sites of replication for WoDV and other DLAs is crucial to better understand the authentic and physiological host response. Thus, a definitive conclusion regarding the innate immune activation by DLAs in their respective host cells cannot be drawn so far.

It has been shown that viral proteins can modulate host factors involved in innate immunity regulation and signaling^44–46^. For instance, hepatitis A virus polyproteins, specifically 3ABC and 3CD, play a role in disrupting Toll-like receptor 3 (TLR3) signaling by targeting the adaptor protein TIR-domain-containing adaptor-inducing IFN-β (TRIF). Additionally, the non-structural protein ORF1 of hepatitis E virus has been shown to inhibit RIG-I/MDA5-mediated IFN-β signaling and NF-κB activation, thereby antagonizing the innate immune response in infected cells^46^. We therefore investigated whether the small delta antigens of WoDV and DeDV possibly interfere with IFN signaling, especially with respect to our finding that they show marked differences in their localization in infected HepaRG^NTCP^ cells (Fig.S5). While HDV S-HDAg predominantly localizes in the nuclei of infected cells, WoDV and DeDV DAgs locate in both the cytosol and the nuclei. This could facilitate countermeasures against sensing and mounting of an effective innate immune response. However, our observation that the innate immune induction upon IFN stimulation remained unchanged in cells expressing WoDAg or DeDAg (Fig.S6) indicated that they did not abrogate the IFN signaling pathway. Nevertheless, we cannot exclude a protective function for the DAg by shielding viral RNA and thereby preventing degradation and MDA5-mediated sensing.

During HDV replication, L-HDAg is expressed following editing of antigenomic HDV RNA by the host enzyme ADAR1^47^. *Hartwig et al.*. demonstrated that the activity of the IFN-induced ISG ADAR1 could enhance editing on the antigenomic HDV RNA. This changes the UAG stop codon, which terminates the S-HDAg ORF, to a tryptophan codon, resulting in a 19/20 amino acid prolonged L-HDAg. Its modification by farnesyltransferase enables packaging into HBV envelopes but also weakens HDV replication^48,49^. We previously demonstrated no enhancement in HDV genome editing following IFN treatment at late stages of viral HDV replication^2^ indicating that IFN-induced ADAR1 might not play a decisive role in L-HDAg-mediated inhibition of HDV replication. Using an HDV variant defective for L-HDAg expression (Fig.S7), we found that CDMS was similarly inhibited by self-induced endogenous IFNs in infected HepaRG^NTCP^ as well as by exogenous IFN treatment in HuH7^NTCP^ (Fig. 6), suggesting that the absence of L-HDAg does not affect the susceptibility of HDV to the IFN response.

In the context of variability in IFN sensitivity, it is important to highlight the study by *Giersch et al.*, which demonstrated that PEG-IFN-α can reduce HDV loads in stably infected human hepatocytes *in vivo*, with a strain- and isolate-specific response^50^. The identification of virus-specific determinants of IFN-α responsiveness underscores the need to evaluate different HDV strains, as well as DLAs clones, to better understand both the virological and host-mediated mechanisms of HDV responsiveness to IFN.

Taken together, our results show that in contrast to HDV, WoDV and DeDV replicate and spread via cell division in human and woodchuck hepatoma cell lines independently of the antiviral state of the infected cells. Most importantly, exogenous IFN treatment did not significantly affect the cell division-mediated spread of WoDV and DeDV in infected HuH7^NTCP^ cells. Moreover, WoDV was able to spread upon clonal expansion even in IFN-stimulated woodchuck-derived cells, shedding light on the still unknown evasion mechanism employed to counteract innate immune responses.

We acknowledge that WoDV and DeDV are not yet established as naturally infectious viruses. Their natural envelope proteins and host reservoirs remain unknown, and infectivity in natural hosts has not been demonstrated. Our pseudotyping system provides an artificial but useful model to study replication and immune interactions, but definitive conclusions require future studies in relevant host cells. Nevertheless, the established cell culture system provides a basis for investigating the nature of DLAs and contributes to a better understanding of the evolution, spread, and interaction with the innate immune system of this unique group of viral agents.

## Materials and methods

### Cell lines

HEK293T cells (Human embryonic kidney cell line^51^,, HuH7 (Human hepatocarcinoma cells^52^, without and with NTCP-overexpression^8^ and woodchuck hepatoma-derived WCH17 (kindly provided by Dr. Carla Coffin, University of Calgary, Canada) cells were cultured using Dulbecco Modified Eagle Medium (Gibco, Cat:41965-039) supplemented with 10% heat-inactivated fetal bovine serum (Sigma-Aldrich, Cat: S0615), 0.1% Penicillin-Streptomycin (Gibco, Cat: 15140122) and 0.1% L-Glutamine (Gibco, Cat: 25030081). For WCH17 cells, 50 µg/mL Blasticidin (InvivoGen, Cat: antbl-05) was added additionally to the medium for antibiotic selection of NTCP. NTCP-overexpressing human hepatoma cells HepaRG^NTCP^ (Human hepatic cell line^8^, were maintained in Williams E medium (Gibco, Cat: 12551032) supplied with 10% heat-inactivated Cytivia HyClone FetalClon II Serum (Fisher Scientific, Cat:10326762), 0.1% Penicillin Streptomycin (Gibco, Cat: 15140122), 0.1% L-Glutamine (Gibco, Cat: 25030081), 4 µg/µL Insulin (Sigma-Aldrich, Cat:91077 C-1G) and 200 µg/µL Hydrocortison (SigmaAldrich, Cat: H4881).

### Pseudoparticles production and purification

For the production of HDV and DLAs pseudoparticles, human hepatoma cells (HuH7) cells were seeded at a density of 5 × 10^6^ per 10-cm dish and the day after they were transfected with 2.5 µg of HDV or DLA plasmids, generated and characterized in our previous work^27^ and 2.5 µg of a plasmid expressing HBV envelope proteins pT7HB2.7 (genotype D^30^, using LT1 Transfection reagent (prod. No. MIR 2305). Twenty-four hours post -transfection (pt), cells were washed with PBS once and medium was replaced. For DLAs pseudoparticles production, three days pt, cells were transfected a second time with a plasmid encoding for the human HDV L-HDAg, to allow packaging of viral RNA by HBsAg. Supernatants were collected, pooled and applied to heparin affinity chromatography using a 5-ml heparin-Sepharose column. The viral particles were eluted, and pseudoparticles-containing fractions were pooled, mixed with distilled water and FCS (final 10%), aliquoted and stored at −80 °C.

### Cell division-mediated viral amplification assay

Different cell lines were infected with HDV and DLAs pseudoparticles. On day 6 post infection (pi) cells were split at different dilution factors depending on the cell line used, to allow clonal expansion. After confluence was reached, cells were fixed, and DAg expression was visualized by immunofluorescence (IF) analysis using a monoclonal antibody raised against S-HDAg (FD3A7, Kerafast, Cat: EHD001).

### Interferon treatment in HuH7^NTCP^ cells

Innate immune system stimulation was initiated via IFN-α or IFN-λ treatment. Cells were incubated with medium containing IFN-α2A (PBL Assay science, Cat:11100-1) or IFN-λ1 (PeproTech, Cat: 300–02 L-20) at a final concentration of 200 IU/mL or 10 µg/ml respectively, for 24 h or during clonal expansion. Cells were harvested and lysed for intracellular RNA quantification via RT-qPCR or fixed after expanding cells reached confluency.

### Innate immunity stimulation in WCH17 cells

Using a 24-well plate, 3 × 10^5^ cells HuH7^NTCP^ and WCH17^NTCP^ cells were seeded per well. The cells were treated with 200 IU/mL IFN-α2A (human) (PBL Assay science, Cat:11100-1), 500 IU/mL IFN-αA/D (pan-species IFN) (Sigma, Cat:14401) or 0.1 µg polyinosinic-polycytidylic acid (Poly I: C). In addition, 0.1 µg Poly I: C was transfected into the cells using Lipofectamine 3000, following manufacturer’s instructions. For ISGs assessment after stimulation, cells were collected at 2-, 12- and 24-hours post-treatment/transfection. Intracellular RNA was extracted following the manufacturer’s instructions of the Nucleospin RNA kit.

For further details regarding materials and methods, please refer to the supplementary information.

## Supplementary Information

Below is the link to the electronic supplementary material.

Supplementary Material 1

## Data Availability

All data generated or analyzed during this study are included in this published article and its Supplementary Information files.

## Abbreviations

ADAR: adenosine deaminase acting on RNA
CDMS: cell division-mediated spread
BLV: Bulevirtide
DAg: delta antigen
DeDV: deer delta agent
FCS: fetal calf serum
GAPDH: glyceraldehyde-3-phosphate dehydrogenase
HBsAg: hepatitis B virus surface antigen
HDAg: hepatitis delta antigen (S, small, L,large)
DLAs: HDV-like agents
IFNs: interferons (α,alpha,β,beta,λ,lambda)
ISGs: interferon stimulated genes
JAK 1 and 2: Janus kinase 1 and 2
LGP2: laboratory of Genetics and Physiology 2
LLoD: lower limit of detection
MDA5: melanoma differentiation-associated protein 5
MGE: multiplicity of genome equivalents
MOI: multiplicity of infection
Mx1: myxovirus resistance gene 1
NF-κB: nuclear factor kappa B
NTCP: sodium (Na+) taurocholate co-transporting polypeptide
PAMPs: pathogen-associated molecular patterns
PEG: polyethylene glycol
PEG-IFNα: pegylated interferon alpha
pi: post infection
pt: post transfection
PRR: pattern recognition receptor
poly I:C: polyinosinic-polycytidylic acid
RIG-I: retinoic acid-inducible gene I
RNP: ribonucleoprotein
RSAD2: radical S-adenosyl methionine domain-containing protein 2
RT-qPCR: reverse transcription quantitative PCR
TLR: toll-like receptor
TRIF: TIR-domain-containing adapter-inducing interferon-β
WoDV: woodchuck delta agent
WT: wild type
