# Supplementary material for: Woodchuck and deer Hepatitis Delta-like agents show distinct innate immune activation and IFN-resistance compared to human Hepatitis D Virus
Source: Sci Rep. 2026 Apr 1;16:11230. doi: 10.1038/s41598-026-45998-w (PMC13046733; doi:10.1038/s41598-026-45998-w)
Supplement: Supplementary file 1 — Supplementary Material 1 [file 41598_2026_45998_MOESM1_ESM.docx]

**“Woodchuck and deer Hepatitis Delta-like agents show distinct innate immune activation and resistance compared to human HDV”**

### Gnimah Eva Gnouamozi ^1^, Annick Charlotte Kooij ^1^, Marie Rose Schrimpf ^1^, Benno Zehnder ^1,3^, Zhenfeng Zhang ^1,2^, Stephan Urban ^1,3^ **^*^**

**Affiliations:**

**^1^ Heidelberg University, Medical Faculty Heidelberg, Department of Infectious Diseases, Molecular Virology, Heidelberg, Germany**

**^2^ School of Public Health and Emergency Management, School of Medicine, Southern University of Science and Technology, Shenzhen, China**

**^3^ German Center for Infection Research (DZIF), Partner Site Heidelberg, Heidelberg, Germany**

*** Corresponding author:** Prof. Dr. Stephan Urban ; Email: Stephan.Urban@med.uni-heidelberg.de

**Supplementary Figures**


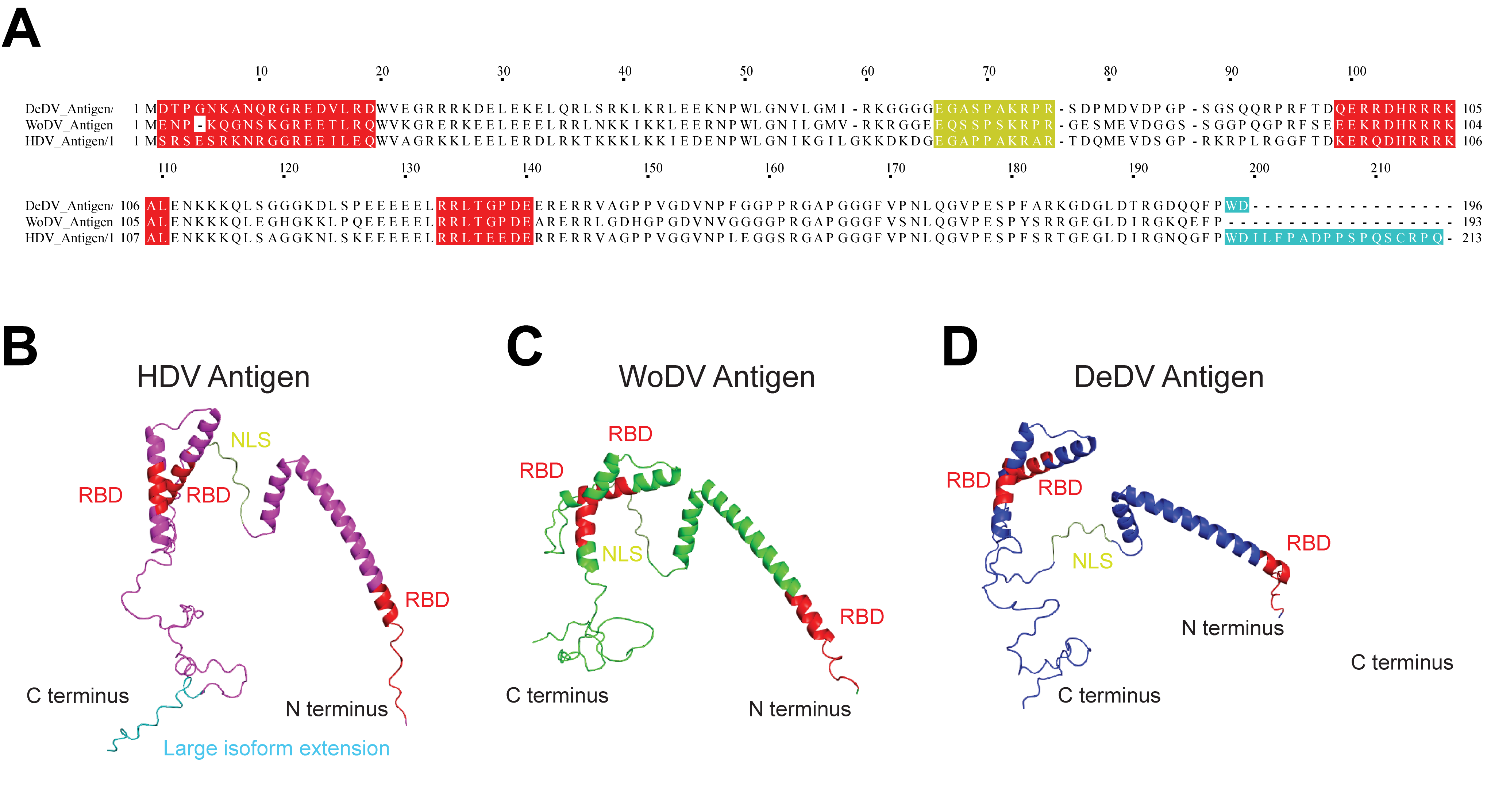


**Figure S1**. **Comparison of HDV and HDV-like agents antigens.** (A) Alignment of the large delta antigen sequences of HDV and HDV-like agents. The translated genome of human HDV is compared with the putative S-DAg of WoDV and DeDV. The translations of the viral proteins were aligned using MUSCLE and visualized using Jalview. Highlighted in the alignment sequence are the RNA binding domains (RBD, red), the nuclear localization signal (NLS, smudge green) and putative large delta antigen extension (light blue) generated by ADAR1 editing of UAG stop codon present in HDV and DeDV sequences. Prediction of secondary structure of HDV (B), WoDV (C), DeDV (D) antigens generated using Alpha Fold software.


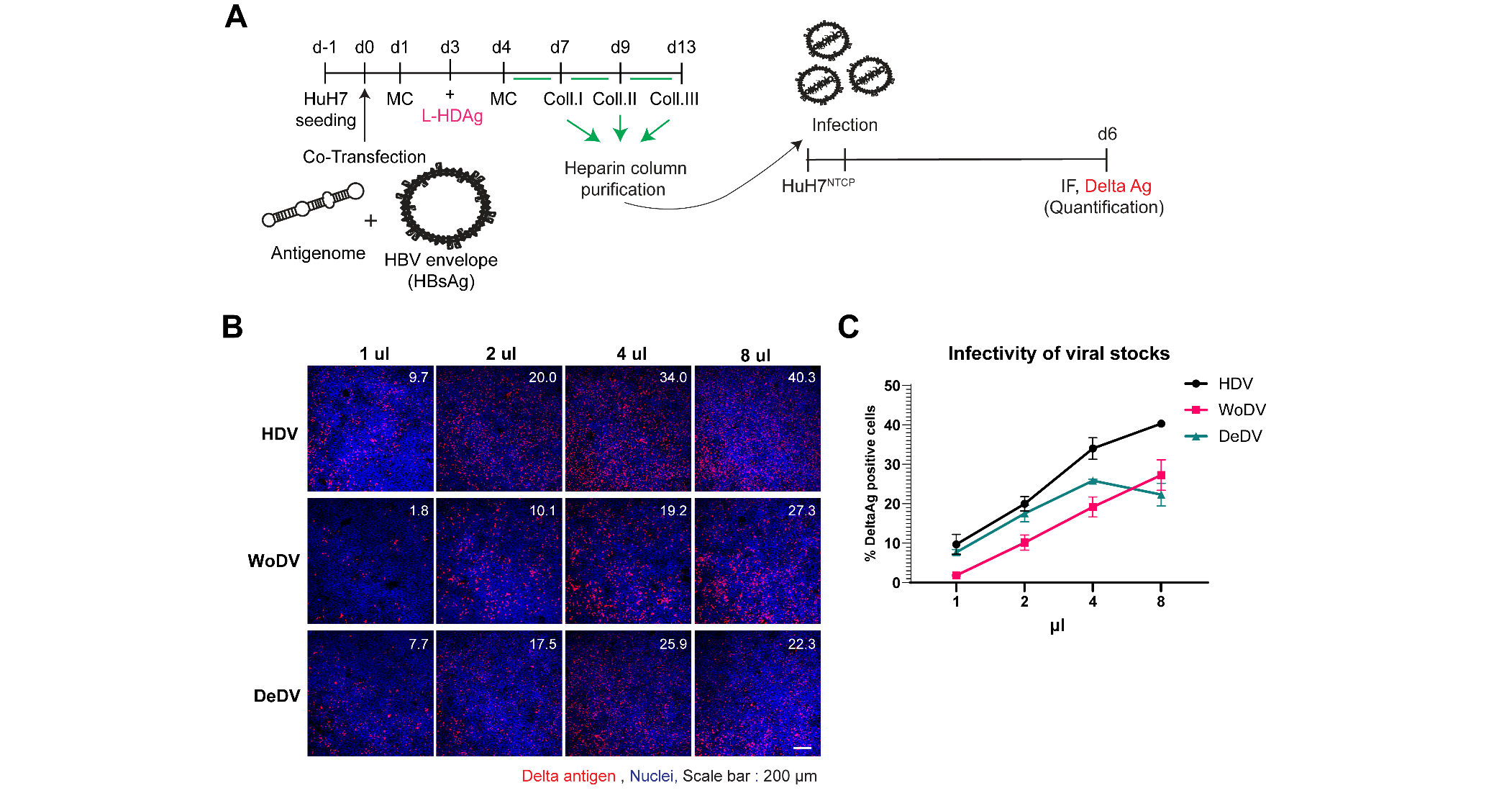


**Figure S2. L-HDAg complementation and packaging of WoDV and DeDV RNPs by HBV envelope proteins.** HuH7 cells were co-transfected with DLAs 1.1 mer antigenome constructs and pT7-HB2.7 plasmid with L-HDAg trans-complementation. Supernatant from transfection was collected, and after heparin column purification, the infectivity of the virus stock was assessed via infection of HuH7^NTCP^ cells (A). 6 days post-infection, cells were fixed and stained for DAg visualization (B). DAg-positive cells were quantified using Ilastik program and are shown as percentages (C). Values are shown as mean ± SD, n = 2. Scale bar: 200 µm.


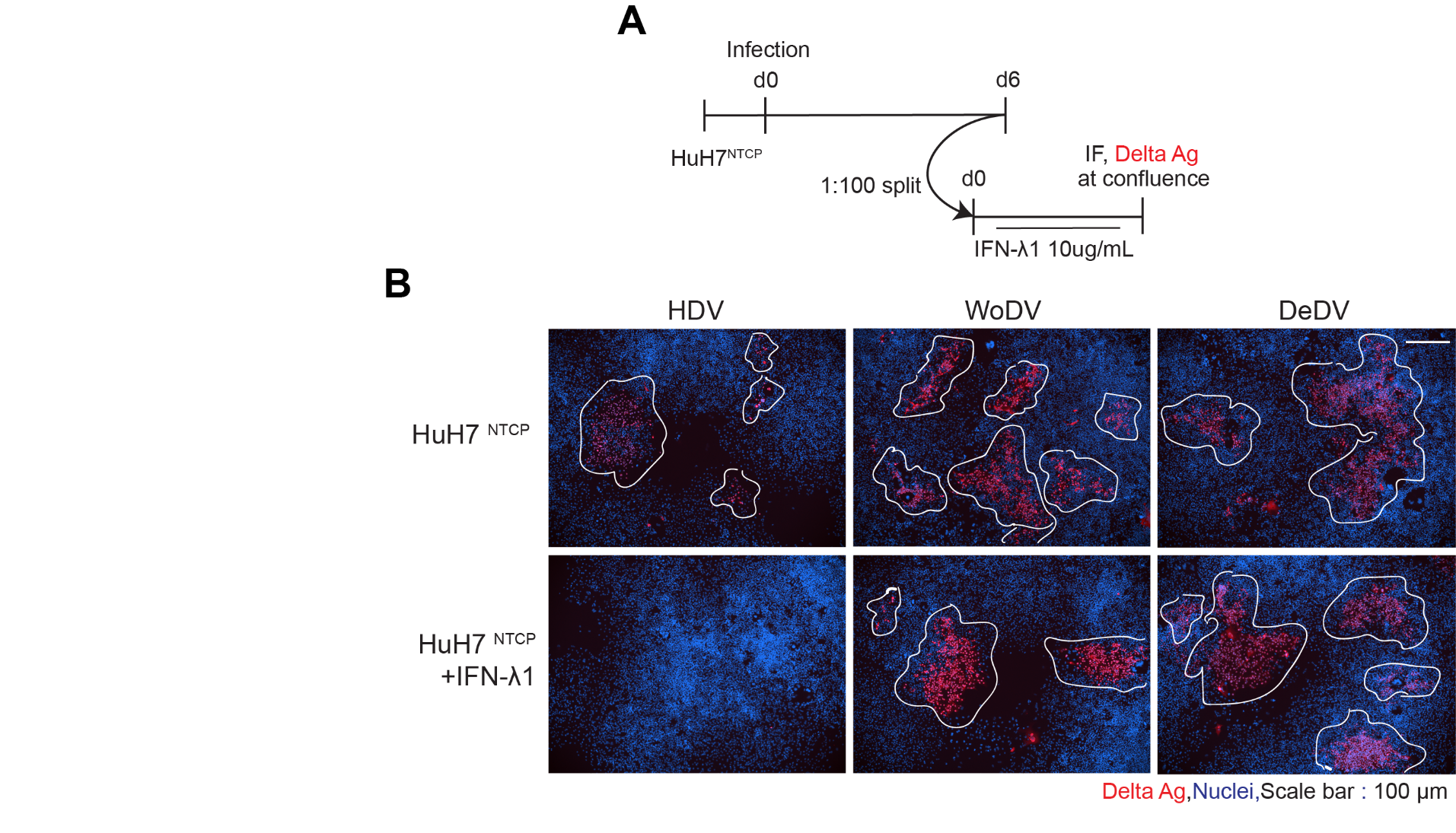


**Figure S3. Cell division-mediated spread of WoDV and DeDV in infected HuH7 ^NTCP^ cells during exogenous IFN-λ1 treatment**. HuH7^NTCP^ cells were infected with HDV, WoDV, and DeDV pseudoparticles and passaged (1:100 dilution) at day 6 pi (A). Cells were then treated with 10 µg/ml of IFN-λ1 until confluence was reached. DAg-positive cells (red) were visualized by IF staining (B). Scale bar: 100 µm.


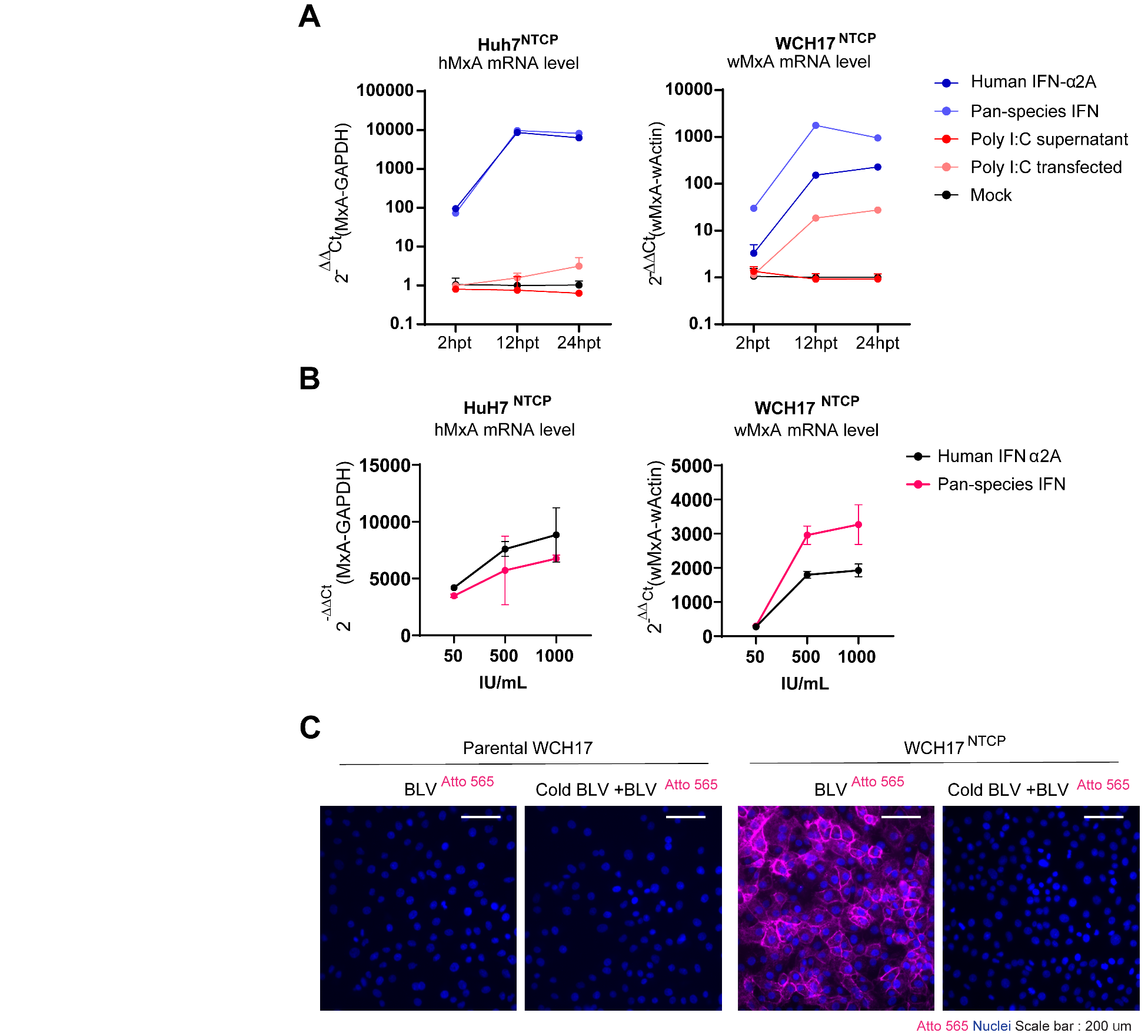


**Figure S4**. **Characterization of innate immune stimulation in WCH17^NTCP^ cells and transduction for stable NTCP expression.** WCH17^NTCP^ and HuH7^NTCP^ cells were treated with human IFN-α2A, pan-species IFN-αA/D and poly I:C or transfected with poly I:C. At 2, 12 and 24 hours post-treatment/transfection, human MxA for HuH7^NTCP^ cells and woodchuck MxA for WCH17^NTCP^ cells mRNA levels were assessed using RT-qPCR. mRNA level was normalized to human GAPDH for HuH7^NTCP^ cells and to woodchuck actin (wActin) for WCH17^NTCP^ cells and displayed as the fold change relative to non-treated/transfected cells. Values are shown as mean ± SD n=2 (A). WCH17^NTCP^ and Huh7^NTCP^ cells were treated with human IFN-α2A, pan-species IFN-αA/D using increasing concentration (50 , 500 or 1000 IU/ml). 12 hours post-treatment, human MxA for HuH7^NTCP^ cells and woodchuck MxA for WCH17^NTCP^ cells were measured for determination of innate immunity induction (B). Parental WCH17 cells were stably transduced using a pWPI NTCP plasmid and after selection, NTCP expression was validated via peptide binding. Parental and newly generated WCH17^NTCP^ cells were incubated with Bulevirtide (BLV) coupled to an ATTO565 fluorescent dye. As a binding specificity control, cells were additionally pre-incubated with BLV alone before incubation with BLV-ATTO 565 (C). Scale bar: 200 µm.


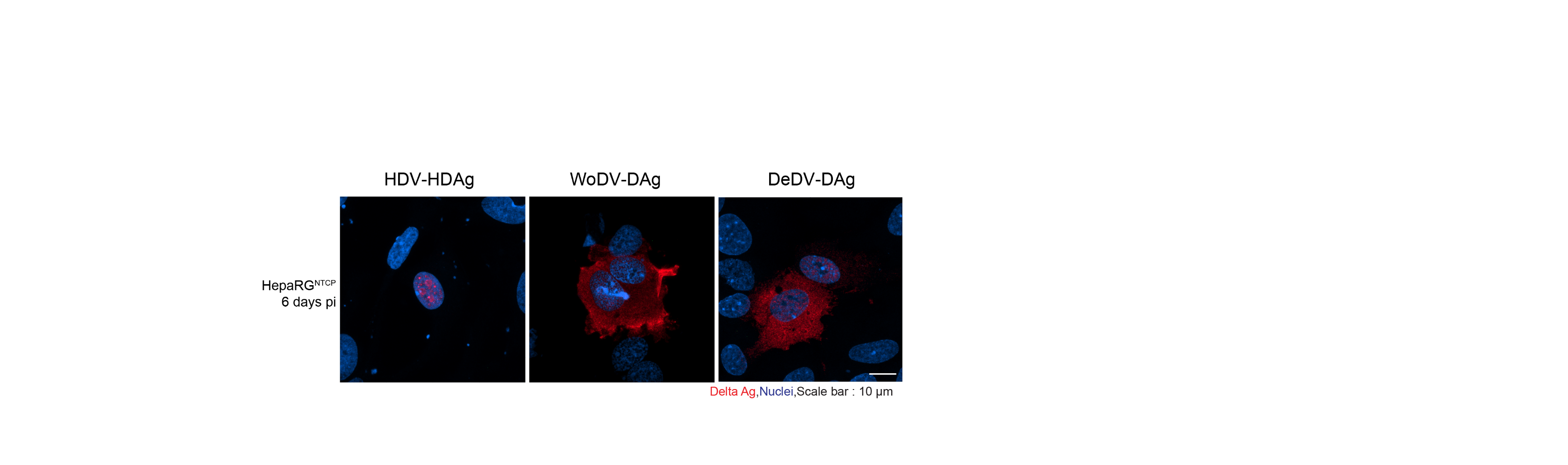


**Figure S5. Subcellular localization of WoDV and DeDV antigens in infected HepaRG^NTCP^ cells.** HepaRG^NTCP^ cells were infected with HDV, WoDV, and DeDV pseudo particles and 6 days pi, cells were fixed to stain for DAg. Samples were imaged using a Confocal microscope (Zeiss Airyscan 2) to visualize subcellular antigen localization. Scale bar: 10 µm.


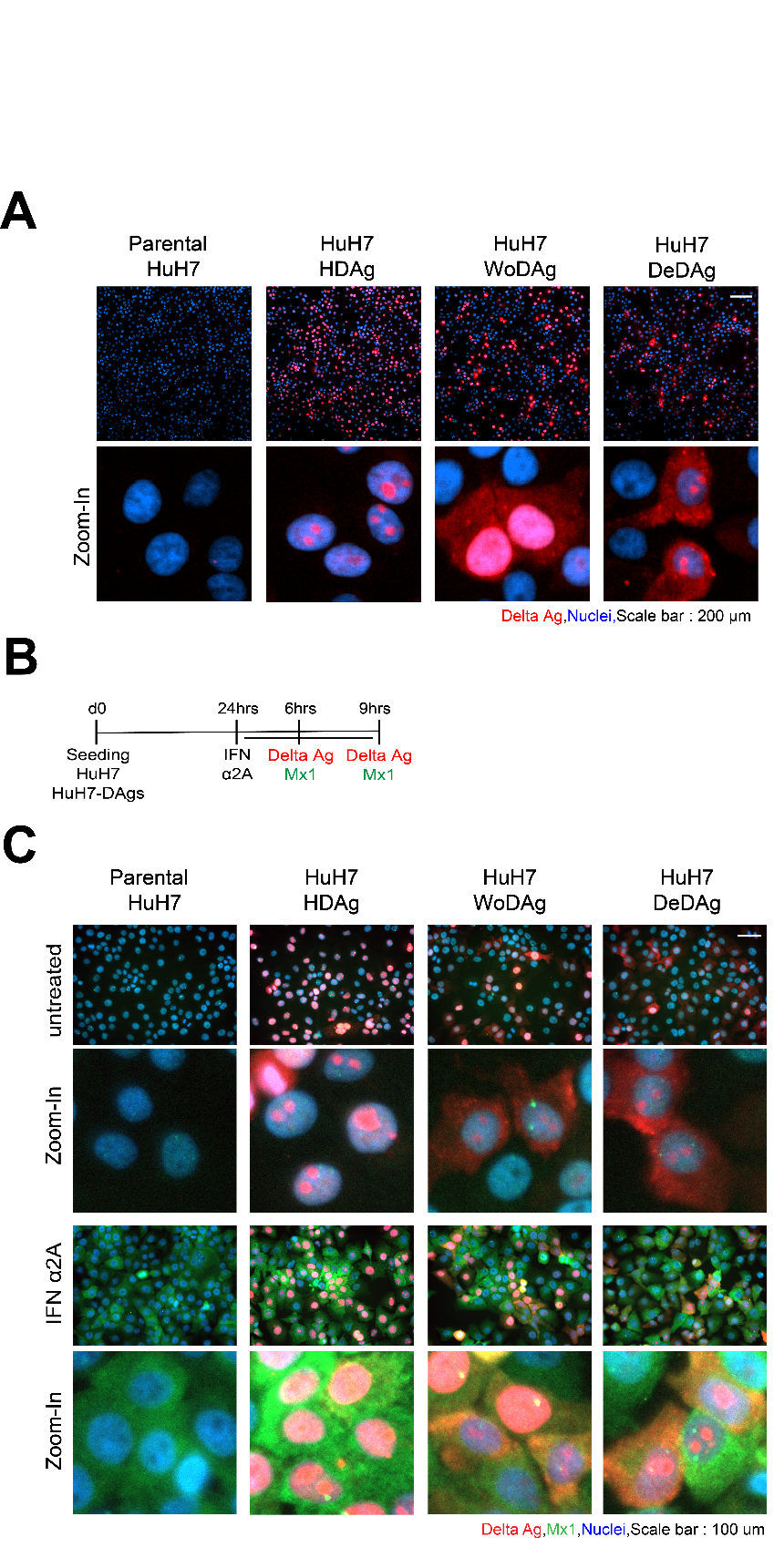


**Figure S6**. **WoDV and DeDV DAg do not significantly affect ISG induction upon interferon treatment**. HuH7 were transduced with lentiviral vectors encoding HDAg, WoDAg or DeDAg and after antibiotic selection, the expression of the different DAgs was assessed via IF staining. Scale bar: 200 µm (A). All cell lines were treated with IFN-α2A for 6 or 9 hours (B). At selected time point (6hrs) cells were fixed for Mx1 visualization via IF analysis. Scale bar: 100 µm (C).


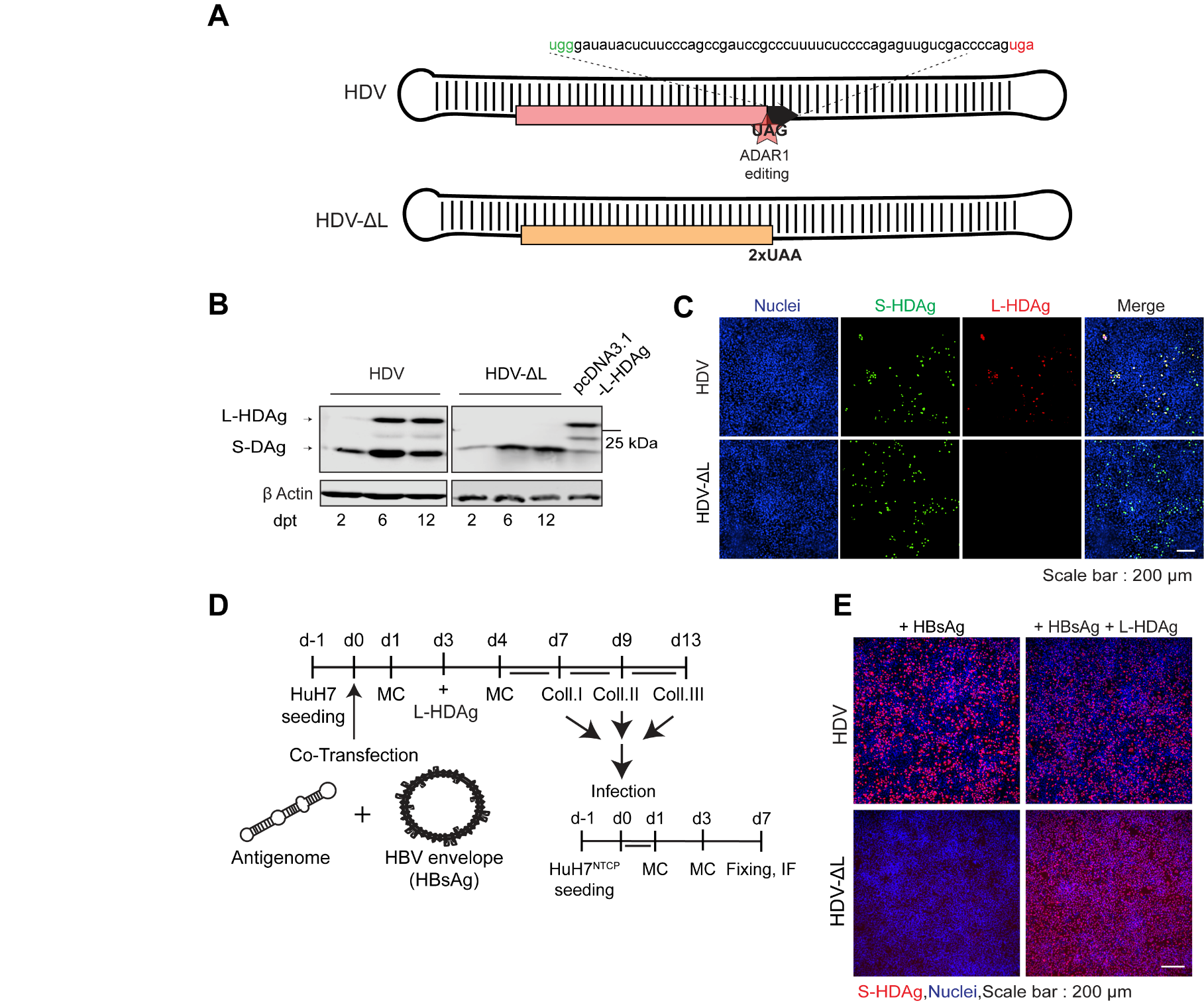


**Figure S7. Characterization of HDV L-HDAg deficient mutant and packaging by HBV envelope proteins.** (A) Schematic representation of HDV edited genome and HDV-ΔL, lacking ADAR1 editing site. HuH7 cells were transfected with HDV WT and HDV-ΔL antigenome constructs, and after 2, 6 and 12 days (dpt), antigen expression was evaluated via western blot (B). Transfected cells were also fixed 7 dpt and stained using antibodies against S- and L-HDAg (C). HuH7 cells were co-transfected with HDV WT and HDV-ΔL 1.1mer antigenome constructs and pT7-HB2.7 plasmid with L-HDAg trans-complementation (D). Supernatant from transfection was collected, and after heparin column purification, the infectivity was assessed via infection of HuH7^NTCP^ cells (E). Scale bar: 200 µm.


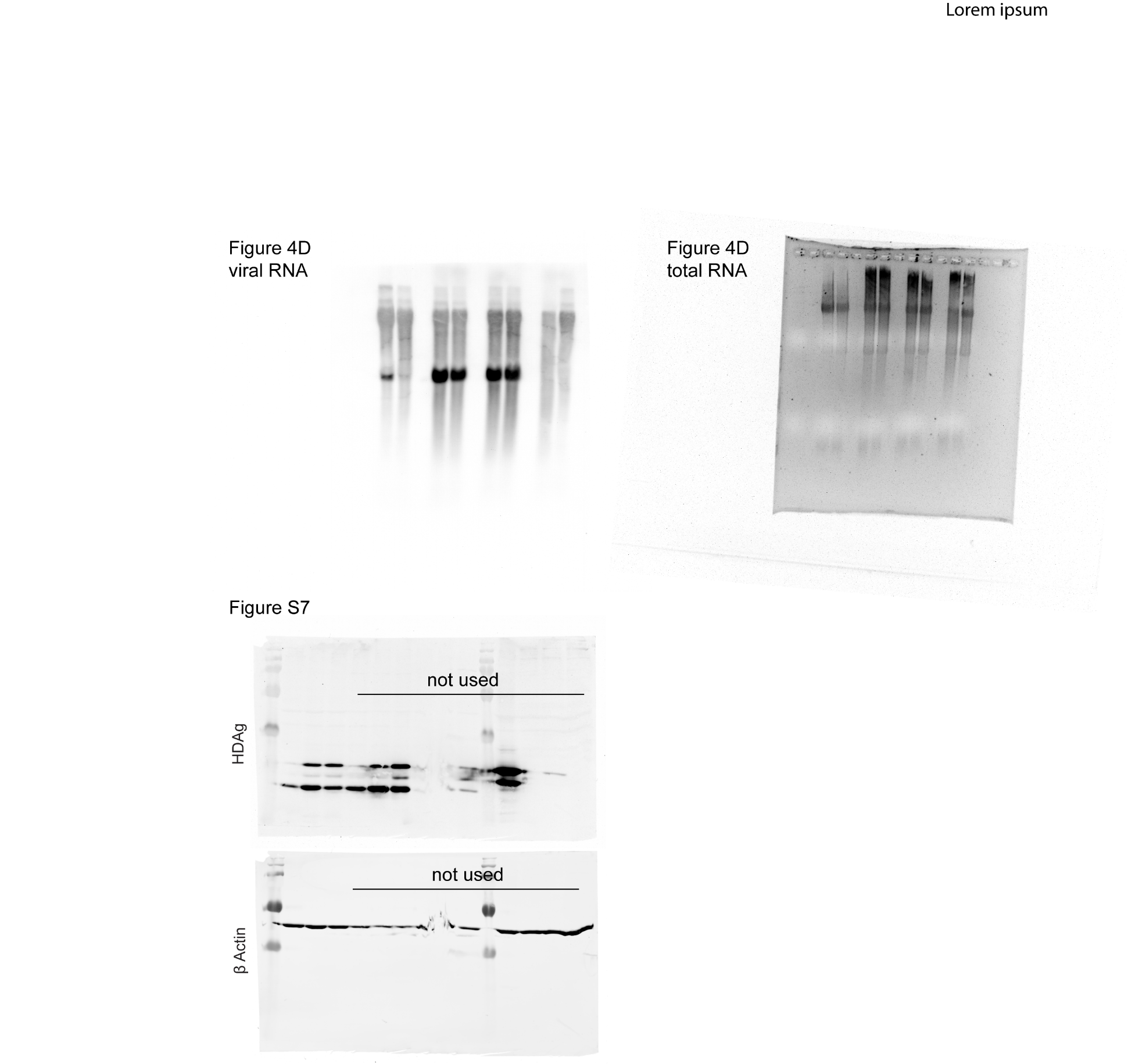


**Figure S8. Original and uncropped blots contained in the manuscripts.** Subfigures are labeled according to their appearance in main manuscript.


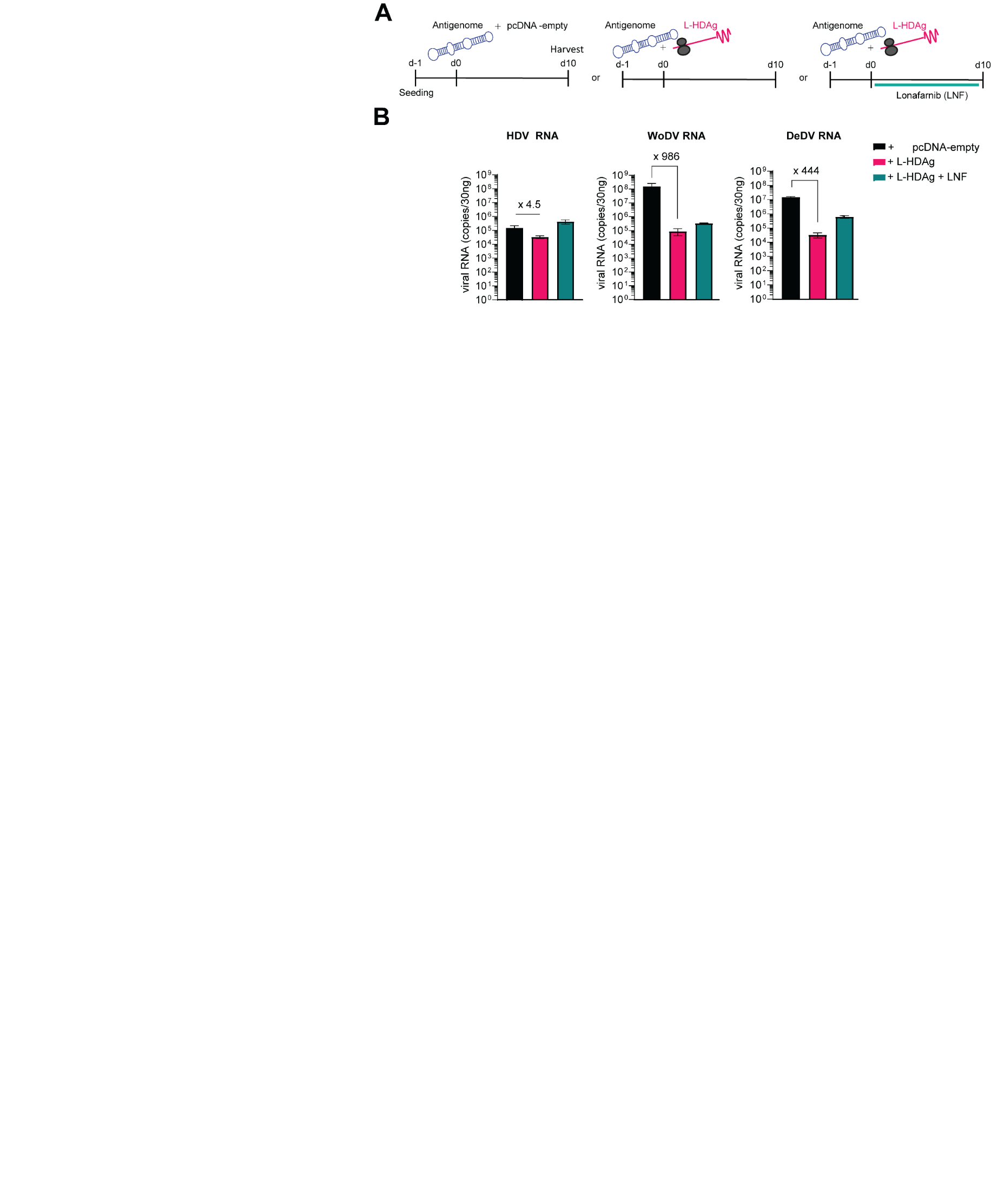


**Figure S9. WoDV and DeDV replication are inhibited by exogenous expression of HDV large delta antigen.** (A) HuH7 cells were transfected with HDV,WoDV and DeDV 1.1x mer antigenome constructs together with a plasmid encoding for the L-HDAg of the human HDV. In parallel, Lonafarnib (LNF) treatment was performed to prevent L-HDAg farnesylation. (B) 10 dpt, viral RNA levels were quantified via RT-qPCR using specific primers for each agent. Data represent mean ± SD of biological duplicates with technical duplicates.

**Figure S10. ISGs upregulation in infected HepaRG NT KO and MDA5 KO cells**. HepaRG^NTCP^ NT KO and HepaRG^NTCP^ MDA5 KO cells were infected in the absence or presence of BLV. Infected cells were harvested at day 6 pi and RSDA2 mRNAs level as well as HDV and DeDV RNA were quantified using RT-qPCR. Data are normalized to GAPDH and displayed as fold change to uninfected condition. Data represent mean ± SD of biological duplicates with technical duplicates.

**Supplementary materials and methods**

**Virus purification using heparin column chromatography**

Supernatant from transient transfection of HuH7 cells, was collected, pooled and applied to heparin affinity chromatography using a 5-ml heparin-Sepharose column: the column was equilibrated with 3 column volumes (CVs) TN buffer at 2 ml/min, followed by sample application at 1 ml/min. Unbound sample was washed out from the column with 5 CVs TN buffer (2 ml/min). The viral particles were eluted from the column with a linear gradient of TN2140 over 10 CVs, during which fractions of 2.5 ml were collected. The column was re-equilibrated with a 5 CVs TN buffer. Virus-containing fractions were pooled (7.5 ml in total), mixed with ddH2O and FCS, aliquoted and stored at -80 °C.

**Infection of NTCP-expressing cell lines**

For infection experiments, cells were seeded in 24-well plates at a concentration of 2.5 x10^5^. The following day, infection medium was prepared containing purified virus at MOI of 1, DMSO, and PEG8000 (4% final concentration). After 16 hours cells were washed twice with PBS, and medium was changed with DMSO-containing medium at day 1 and 3 post-infection (pi). As an entry inhibition control, the cells were treated with 200 nM BLV, 15 minutes prior to and during infection. At designated time points, cells were washed 3 times with PBS and fixed for immunofluorescence (IF) analysis.

**RNA purification of intracellular RNA**

Intracellular RNA extraction from harvested cells was carried out using the NucleoSpin RNA Kit following the manufacturer’s instructions. RNA concentration in each sample was measured by Nanodrop.

**Reverse transcription (RT) and Quantitative PCR (RT-qPCR) for intracellular mRNA detection**

Reverse transcription was performed according to the High-capacity cDNA reverse transcription kit. The obtained cDNA was diluted 1:2.5 with Braun water for RT-Quantitative PCR (qPCR) analysis. RT-qPCR was performed using iTaq universal SYBR Green Supermax on a CFX96 thermocycler as indicated by manufacturer’s instructions. Copy numbers were quantified using pJC126 plasmid for HDV or plasmids containing the respective HDV-like agent genome. For ISGs detection, the values for each gene were normalized to GAPDH as housekeeping gene.

**Northern Blot**

RNA extraction was performed as mentioned above. The total RNA was then quantified using Nanodrop and diluted to achieve the same final RNA concentration. A total of 10 µg RNA/sample was loaded on an agarose/paraformaldehyde (PFA) gel. RNA was run in a 1.5% MOPS agarose gel containing 2.2M formaldehyde. After denaturation (50mM NaOH for 5min), RNA was transferred to a nylon membrane by capillary transfer using 20× SSC buffer. Membranes were dried and fixed by UV crosslinking. Virus-specific probes were synthesized via *in vitro* transcription (IVT) using the Digoxigenin (DIG) RNA labeling Mix. Membranes were hybridized at 60℃ overnight with a mixture of 3 different probes (against HDV, WoDV or DeDV genomes). The following day the probe mix was removed, and the membrane was washed 2 times with 20 ml 2X SSC/0.1% SDS for 5min at RT. After 2 additional washes with 20 ml 0.2X SSC/0.1% SDS, for 15-20 min at 60℃ in the hybridization oven, the membrane was washed with 20 ml 1×DIG-wash buffer for 5min at RT. The membrane was then blocked with 20 ml blocking solution for 30 - 60min at RT and incubated with antibody solution for 1h. After 3 washes with 20 ml 1xDIG wash buffer (3x10 min at RT), membrane was equilibrated with 15 ml 1×detection buffer for 2 - 5min incubated with detection solution for 10 - 30 min at 37℃. Signal was detected by INTAS instrument, 1 min for 30 sequential acquisitions.

**Generation of stable cell lines**

Woodchuck hepatoma (WCH17) cells were kindly provided by Dr. Carla Coffin, University of Calgary, Canada. NTCP-overexpressing WCH17 cells were made using lentiviral transduction. Using 10 cm dishes, 3.5x10^6^ HEK293T cells were seeded per dish. The cells were transfected with 9 µg of psPAX2, 6 µg of pMD2.G and 9 µg of pWPI-Blasticidin plasmid containing NTCP the day after seeding using polyethyleneimine (PEI) (Sigma-Aldrich, Cat:40,872-7). 24 hours post transfection, the medium was changed. The lentiviruses in the supernatant were harvested at 48 and 72 hours after transfection. After centrifugation (5 min, 500 rpm) and filtration through a 0.45 µm syringe filter (Millipore, Cat: SLHA03355), the lentiviruses were added to WCH17 cells together with 4% Polyethylene glycol (PEG) (Sigma-Aldrich, Cat:89510). 24 hours after transduction the medium was changed. The transduced WCH17 cells were transferred to a T25 flask 72 hours post-transduction. For the NTCP antibiotic selection, 50 µg/mL Blasticidin (InvivoGen, Cat: ant-bl-05) was used. To evaluate NTCP transduction efficiency, the cells were incubated with 200 nM of Bulevirtide (BLV)-ATTO565 peptide for 15 min at 37° C. As specificity control, some wells were pre-incubated with unlabelled BLV. HuH7^NTCP^ cells were stained simultaneously as comparison control. After peptide incubation, the cells were washed twice with 2% BSA-PBS, fixed using 1.25% PFA. The nuclei were stained using 2 µg/mL Hoechst 33342 (Invitrogen, Cat:H3570).

HuH7 cells stably expressing WoDV, DeDV or HDV (S)DAg (HuH7/WoDAg, HuH7/DeDAg, HuH7/HDAg) were generated as described above. pWPI-Puro plasmids containing HDV, WoDV or DeDV (S)DAg were used for transfection in HEK293T cells. Antibiotic selection for DAg expression was carried out using 5 µg/mL Puromycin (InvivoGen, Cat: ant-pr-1).

**Immunofluorescence**

Cells were washed once with PBS, then fixed with 4% PFA at RT for 20 min. After three washing steps with PBS, permeabilization buffer (Triton X 100, 0,5%) was added, the cells were permeabilized at RT for 10 min and washed thrice with PBS. The cells were incubated with primary antibody at the proper dilution in 2% BSA/PBS at RT for 1 hour. After three washing steps with PBS, cells were incubated with 1:1000 diluted secondary antibody and 2 µg/mL Hoechst 33342 stain for 1 hour at RT while shaking, protected from light. Cells seeded on coverslips were stained as described above. Then, the coverslips were washed in PBS and mounted on glass slides with 10 µL of Fluor mount-G mounting medium. DAg expression was checked with IF using 1:3000 diluted rabbit anti-DAg (FD3A7) (Kerafast, Cat: EHD001) as a primary antibody and 1:1000 diluted goat anti-rabbit AlexaFluor 546 (Thermo Scientific, Cat:A11010) as secondary antibody.

The cells were imaged using Widefield Zeiss Cell Discoverer 7 microscope (10x or 20x magnification). For sub-cellular localization, images were taken at 40x magnification using Confocal Microscope Zeiss Airyscan 2. Both microscopes belong to the
Infectious Diseases Imaging Platform (IDIP) Heidelberg facility.

**Western blot**

For western blot analysis, cells were cultured in 24-well plates until the desired timepoint. Cells were washed once with PBS, then lysed in 75 µL 2x SDS sample buffer. The lysates were stored at -20°C. Before loading the SDS gel, the samples were vortexed, heated to 95°C for 10 min, and centrifuged at maximum speed for 20 minutes. For SDS PAGE, a resolving gel containing 15% polyacrylamide was combined with a 3% stacking gel. For each well, 3 μl of lysate was added. After resolving in SDS running buffer at 60 V for 20 min then at 120 V, the proteins were transferred from the gel to a nitrocellulose membrane using a semi-dry system at 25 V for 30 min. Directly after blotting, the membrane was incubated in blocking buffer for 1 hour at RT. The membrane was incubated with primary antibody solution at the appropriate dilution in blocking buffer (1% Casein in PBS) at 4°C, overnight. After washing thrice with TBST for 10 min, 10 mL of the secondary antibodies (goat anti-rabbit 800 and goat anti-mouse 680 both 1:10,000 in blocking buffer) was applied for 1 h at RT, acquisition was performed using LI-COR Odyssey M instrument.

**Generation of HDV-L-minus clone and pseudoparticle**

HDV pJC126 plasmid was subcloned to contain a double UAA stop codon at the 3´- end of the S-HDAg ORF abolishing the expression of L-HDAg (HDV-ΔL). Following transfection of HuH7 cells we analyzed for delta antigen expression by WB and IF. In order to perform infection studies, viral particles were generated by co-transfection of HDV-ΔL with HBsAg and a WT-L-HDAg expression vector trans-complementation.

**Protein prediction using AlphaFold program**

The aminoacidic sequences were analyzed using the online software Alpha Fold 2 (https://colab.research.google.com/github/sokrypton/ColabFold/blob/main/AlphaFold2.ipynb) and output files were analyzed and displayed using PyMOL.

**Phylogenetic analysis**

Aminoacidic sequences in FASTA format were aligned using Multiple Sequence Comparison by Log- Expectation (MUSCLE) online tool (https://www.ebi.ac.uk/Tools/msa/muscle/) and output alignment was visualized and modified using Jalview software.

**Statistical analysis**

Statistical analyses were performed using GraphPad Prism 10.4.1. For the comparison of 2 sample groups, p values were determined by a 2-tailed Student’s t test. The statistical significance was presented as follows: *, p < 0.05; **, p < 0.01; ***, p < 0.001; n.s., not significant.

| **Primer** | **Used for** | **Sequence** |
| --- | --- | --- |
| pWPI-WoDV-DAg-Fw | Cloning WoDLA DAg in pWPI vector | TATAATGGATCCCTCGATGGAGAATCCTAAGC |
| pWPI-WoDV-DAg-Rev | Cloning WoDLA DAg in pWPI vector | TATAATACGCGTTTAGGGGAACTTGCTTTC |
| pWPI-DeDV-DAg-Fw | Cloning DeDLA DAg in pWPI vector | TATAATCCTGCAGGATGGACACGCCCGGTAACAA |
| pWPI-DeDV-DAg-Rev | Cloning DeDLA DAg in pWPI vector | TATAATAACGCGTTGGAAACTGCTGATCTCCTC |
| RSAD2-Fw | qPCR – ISG induction | CGTGAGCATCGTGAGCAATG |
| RSAD2-Rev | qPCR – ISG induction | TCTTCTTTCCTTGGCCACGG |
| hGAPDH-Fw | qPCR – ISG induction | GAAGGTGAAGGTCGGAGTC |
| hGAPDH-Rev | qPCR – ISG induction | GAAGATGGTGATGGGATTTC |
| hMx1-Fw | qPCR – ISG induction | AAGAGCCGGCTGTGGATATG |
| hMx1-Rev | qPCR – ISG induction | GGCGGTTCTGTGGAGGTTAA |
| wMxA-Fw | qPCR – ISG induction | GGAGGGAGGAGAAGAGGAAA |
| wMxA-Rev | qPCR – ISG induction | CTGGAGATGCGGTTGTGAG |
| wActin-Fw | qPCR – ISG induction | TGGAATCCTGTGGCATCCATGAAAC |
| wActin-Rev | qPCR – ISG induction | TAAAACGCAGCTCAGTAACAGTCCG |
| hIFN-λ1-Fw | qPCR – IFN induction | CGCCTTGGAAGAGTCACTCA |
| hIFN-λ1-Rev | qPCR – IFN induction | GAAGCCTCAGGTCCCAATTC |
| hIFN-β-Fw | qPCR – IFN induction | ACCAACAAGTGTCTCCTCCA |
| hIFN- β -Rev | qPCR – IFN induction | AAGCCTCCCATTCAATTGCC |
| HDV-Fw | qPCR – Viral RNA detection | ATGAGCCGGTCCGAGTCGAGGAAGA |
| HDV-Rev | qPCR – Viral RNA detection | TTCTTTCTTCCGGCCACCCACTGC |
| WoDV-Fw | qPCR – Viral RNA detection | CCTGGCTGGGGAACATCCTGGGAAT |
| WoDV-Rev | qPCR – Viral RNA detection | TTCTCCTCGTGGTCTCTTGGACGGG |
| DeDV-Fw | qPCR – Viral RNA detection | TTCTCCTCGTGGTCTCTTGGACGGG |
| DeDV-Rev | qPCR – Viral RNA detection | ATCCGATCTTGGTCTCTTGGCCGGG |

**Table S1. Primers.** Oligonucleotides sequences used in this study**.**
